# Chemical Analysis and Investigation of Antimicrobial and Antibiofilm Activities of *Prangos trifida* (Apiaceae)

**DOI:** 10.3390/antibiotics13010041

**Published:** 2024-01-01

**Authors:** Ljuboš Ušjak, Dejan Stojković, Tamara Carević, Violeta Milutinović, Marina Soković, Marjan Niketić, Silvana Petrović

**Affiliations:** 1 Department of Pharmacognosy, University of Belgrade-Faculty of Pharmacy, Vojvode Stepe 450, 11221 Belgrade, Serbia; ljubos.usjak@pharmacy.bg.ac.rs (L.U.); violeta.milutinovic@pharmacy.bg.ac.rs (V.M.); 2 Department of Plant Physiology, Institute for Biological Research “Siniša Stanković”-National Institute of Republic of Serbia, University of Belgrade, Bulevar Despota Stefana 142, 11000 Belgrade, Serbia; tamara.carevic@ibiss.bg.ac.rs (T.C.); mris@ibiss.bg.ac.rs (M.S.); 3 Natural History Museum, Njegoševa 51, 11000 Belgrade, Serbia; mniketic@nhmbeo.rs; 4 Serbian Academy of Sciences and Arts, Kneza Mihaila 35/II, 11000 Belgrade, Serbia

**Keywords:** *Prangos trifida*, essential oils, GC-FID-MS, dichloromethane extracts, coumarins, LC-MS, antibacterial activity, antifungal activity, biofilm inhibition, congo red assay

## Abstract

Plants of the genus *Prangos* are intensively investigated as potential new sources of bioactive isolated products. In this work, the chemical composition of volatile constituents (essential oils and headspace volatiles) and dichloromethane extracts, as well as antimicrobial and antibiofilm activities of essential oils and MFDEs (methanol fractions of dichloromethane extracts) of *Prangos trifida* from Serbia, were investigated. Volatiles of roots, leaves, stems and fruits, and fatty acids and phytosterols in dichloromethane extracts of roots and fruits were analyzed by GC-FID-MS, whereas coumarins in MFDEs by LC–MS and some isolated coumarins by ^1^H-NMR. Minimum inhibitory concentrations (MICs) and minimum bactericidal concentrations/minimum fungicidal concentrations (MBCs/MFCs) of essential oils and MFDEs were determined against 13 microorganisms. Antibiofilm activity was assessed against four microorganisms. Additionally, congo red and ergosterol binding assays were conducted to elucidate selected mechanisms of antibiofilm action in the case of *Candida albicans*. Total of 52 volatile constituents, 16 fatty acids, eight phytosterols and 10 coumarins were identified. Essential oils demonstrated significant activity, surpassing that of commercial food preservatives, against six tested molds from the *Aspergillus*, *Penicillium* and *Trichoderma* genera, as well as against bacteria *Staphylococcus aureus* and *Bacillus cereus*. Most of the oils strongly inhibited the formation of biofilms by *S. aureus, Listeria monocytogenes* and *Escherichia coli*. MFDEs exhibited noteworthy effects against *B. cereus* and the tested *Aspergillus* species, particularly *A. niger*, and significantly inhibited *C. albicans* biofilm formation. This inhibition was linked to a marked reduction in exopolysaccharide production, while antifungal mechanisms associated with ergosterol remained unaffected.

## 1. Introduction

In an era of growing antimicrobial resistance, it is crucial to discover new antibacterial and antifungal agents [1,2]. Plants are well-known to be rich sources of compounds, which have a wide range of biological activities, including effects on broad spectrum of microorganisms [3], the most researched plant preparations are essential oils and extracts [4]. Their extensive chemical characterization is a fundamental requirement in the initial phases of antimicrobial activity research. This approach not only makes it easier to identify preparations with possible antimicrobial abilities, but it also enables understanding of the active components and their potential interactions.

The genus *Prangos* Lindl. (Apiaceae) comprises about 50 species distributed in Eurasia (from Portugal to Tibet), with the center of diversity in the Irano-Turanian region [5,6]. Both underground and aerial parts of various plants of this genus are used in folk medicine. For example, different organs of *P. ferulacea* (L.) Lindl. are used orally for digestive disorders, and as antidiabetic and antihypertensive agents, and externally against parasites and for the treatment of wounds [6,7].

Past phytochemical studies on these plants were dealing primarily with essential oils and coumarins, and to a smaller extent with flavonoids, phenolic acids, *γ*-pyrones, phytosterols, carotenoids and polyacetylenes [6]. For example, recently, the composition of essential oils of leaves and flowers of *P. ferulacea* [8], flowering aerial parts of *P. platychlaena* Boiss. [9], aerial parts and roots of *P. heyniae* H. Duman & M. F. Watson [10,11], and aerial parts of *P. meliocarpoides* Boiss. and *P. uechtritzii* Boiss. & Hausskn. [10], as well as of coumarin-containing extracts of roots of *P. pabularia* Lindl. and *P. hulusii* Şenol, Yıldırım & Seçmen [12,13], and aerial parts of *P. ferulacea*, *P. peucedanifolia* Fenzl [14], *P. heyniae*, *P. meliocarpoides* and *P. uechtritzii* [15], were investigated.

Also, for extracts of different polarity and/or essential oils from various plant parts of *Prangos* species, interesting bioactivities were previously demonstrated. These include in vitro antimicrobial, cytotoxic, antioxidant, enzyme (*α*-amylase, *α*-glucosidase, lipase, tyrosinase, acetyl and butyrylcholinesterase, and angiotensin-converting enzyme) inhibitory and spasmolytic, and in vivo hypoglycemic and analgesic effects [6,7]. It should be noted that the antimicrobial activity is among the most commonly investigated bioactivities of *Prangos* species. For example, it was evaluated for abovementioned *P. ferulacea* and *P. heyniae* essential oils, and for *P. hulusii*, *P. ferulacea* and *P. peucedanifolia* extracts [8,11,13,14]. However, despite this fact, the data on the antibiofilm activities of isolated products of these species are very scarce [6,7]. Namely, the first studies on this topic were published only recently and were focused on the antibiofilm activity of *P. ferulacea* and *P. acaulis* (DC.) Bornm. extracts [16,17].

*Prangos trifida* (Mill.) Herrnst. & Heyn (Figure S1) is sporadically distributed on rocky slopes in European Sub-Mediterranean, from Portugal in the West to Crimea in the East [18,19]. It is a branched glabrous perennial up to 1 m height, with pinnatisect leaves and linear lobes. Fruit umbels are 10–25 rayed, with few small bracts and bracteoles and ovate wingless fruits with thick mesocarp and numerous secretory channels with essential oils [18,20]. More recent molecular studies [21,22] showed separation of two well supported groups: western an eastern clade (including Balkans and Crimea), but with insufficient morphological support. According to some authors [5,20], eastern populations belong to a separate species (under the name *Cachrys alpina* Bieb.).

So far, the composition of essential oils of *P. trifida* fruits from Turkey (plant investigated under the name *C. alpina*) [23], fruits, flowers, stems with leaves and roots from Spain (plant investigated under the name *C. trifida* L.) [24], fruits from Crimea [25], and aerial parts (collected before flowering) from Italy [26,27] were investigated. Essential oil from Italy was also tested for antioxidant [26,27] and antimicrobial activities [26]. Regarding antimicrobial activity, this essential oil was active against *Bacillus cereus* and *B. subtilis*, but not against *Staphylococcus aureus*, *Escherichia coli*, *Pseudomonas aeruginosa* and *Salmonella* Typhimurium (it should be noted that concentrations of the oil of only up to 0.2 mg/mL were tested) [26]. Also, in a search for novel anti-inflammatory agents, for three coumarins, i.e., imperatorin, isoimperatorin and prantschimgin, isolated from *P. trifida* (investigated under the name *C. trifida*), effects on some macrophage functions were demonstrated. Most notably, imperatorin caused strong reduction in nitric oxide generation, imperatorin and isoimperatorin inhibited both cyclooxygenase and lipoxygenase pathways of arachidonate metabolism, whereas prantschimgin showed effect only on the lipoxygenase pathway [28].

In summary, despite there are several studies on the composition of *P. trifida* essential oils, the knowledge on this topic is still insufficient because these essential oils were obtained from the plants collected at only a few localities. Moreover, other *P. trifida* metabolites, as well as bioactivity of isolates of this plant, were very scarcely investigated. Therefore, the aim of the current study was to perform a comprehensive analysis of isolated products obtained from *P. trifida* collected in Serbia. This included chemical analysis, investigation of antimicrobial and antibiofilm activities, and exploration of potential mechanisms of action. We focused on analyzing the compositions of volatile constituents in different plant parts, i.e., roots, leaves, stems and fruits, as well as of dichloromethane (CH_2_Cl_2_) extracts from roots and fruits. Subsequently, we assessed the antimicrobial and antibiofilm activities of the isolates, for which we found relevant metabolites during phytochemical investigations, i.e., the activities of essential oils and coumarin-rich methanol fractions of CH_2_Cl_2_ extracts (MFDEs). Additionally, we screened MFDEs for selected mechanisms of action against *Candida albicans*.

## 2. Results

### 2.1. Chemical Composition of P. trifida

#### 2.1.1. Composition of Essential Oils

The yields of essential oils obtained by hydrodistillation were the lowest in stems (0.10 ± 0.05% *w*/*w*) and roots (0.05 ± 0.01% *w*/*w*), and were not significantly different (*p* = 0.24). Leaves and fruits were richer in essential oils (yields 0.50 ± 0.04% *w*/*w* and 0.37 ± 0.04% *w*/*w*, respectively), and the content of essential oils differed significantly both between these two plant organs and also in relation to the contents of essential oils in stems and roots (*p* < 0.05). The composition of the isolated essential oils was analyzed using GC-FID-MS (Table 1). In this way, 32 components were identified in both the root and leaf essential oils, 39 in the stem oil and 43 in the fruit oil, comprising 95.6, 95.1, 94.0 and 95.2% of the total essential oils, respectively. Dominant constituents of these essential oils were monoterpenes (66.2–87.2%). Monoterpene hydrocarbons, i.e., non-oxygenated monoterpenes, were more abundant in the root (56.2%), leaf (67.2%) and fruit essential oils (66.5%), whereas the stem oil differed due to the prevalence of oxygenated monoterpenes (45.6%). Accordingly, non-oxygenated monoterpenes terpinolene (36.2%) and *p*-cymene (11.5%) were dominant in the root essential oil, (*E*)-*β*-ocimen (23.2%), terpinolene (18.1%) and *α*-pinene (11.3%) in the leaf oil, and *p*-cymene (25.4%), limonene (14.4%) and *γ*-terpinene (11.4%) in the fruit oil. In the stem essential oil, oxygenated monoterpene *p*-cymen-8-ol (21.8%) was the most abundant and it was followed by non-oxygenated compound *p*-cymene (14.1%). Additionally, the stem oil contained a considerable amount of the oxygenated sesquiterpene caryophyllene oxide (13.1%).

#### 2.1.2. Composition of Headspace Volatiles

Headspace volatiles, i.e., the most volatile compounds of investigated *P. trifida* roots, leaves, stems and fruits, were isolated using the headspace sampler and analyzed using GC-FID-MS (Table 1). In total, 15 constituents isolated in this way were identified in the roots, 16 in the leaves, 15 in the stems and 14 in the fruits, representing 100.0, 96.7, 100.0 and 99.7% of the total headspace volatiles, respectively. In all instances, the dominant were monoterpene hydrocarbons (92.3, 93.0, 93.3 and 99.4%, respectively), which was in accordance with their higher volatility compared to oxygenated monoterpenes. α-Pinene (17.3, 21.7 and 19.5% in the leaves, stems and fruits, respectively), sabinene (12.5% in the fruits), *p*-cymene (11.1, 40.3 and 23.3% in the roots, stems and fruits, respectively), limonene (11.6, 21.6 and 23.9% in the roots, stems and fruits, respectively), (*E*)-*β*-ocimene (40.9% in the leaves) and terpinolene (58.5 and 18.6% in the roots and leaves, respectively) were the most abundant headspace volatiles.

#### 2.1.3. Fatty Acid, Phytosterol and Triterpene Composition of CH_2_Cl_2_ Extracts

*Prangos trifida* roots and fruits were extracted with CH_2_Cl_2_. After evaporation of the solvent, semi-solid (oily) extracts were obtained. The yields of the extracts of roots and fruits were significantly different (*p* = 0.03), and amounted to 4.4 ± 0.1% *w*/*w* and 7.6 ± 0.9% *w*/*w*, respectively.

Fatty acids of roots and fruits CH_2_Cl_2_ extracts were analyzed using GC-FID-MS as volatile fatty acid methyl esters (FAME), i.e., after saponification and subsequent methylation; FAME yields: 39.1% *w*/*w* (fruits extract) and 35.5% *w*/*w* (roots extract). Sixteen fatty acids were identified in each sample, comprising 92.6 and 98.7% of all detected fatty acids, respectively (Table 2). Dominant ones in the roots extract were polyunsaturated fatty acids (55.4%), mainly linoleic (51.8%). Notable amounts of saturated palmitic (14.9%) and monounsaturated oleic acid (14.2%) were also revealed. The most abundant in the fruits extract were monounsaturated fatty acids (61.3%), mostly petroselinic (49.9%) and oleic (10.2%). Appreciable amount of linoleic acid (28.3%) was also present.

Yields of residual unsaponifiable fractions were 5.1 and 4.8% *w*/*w* of the roots and fruits extracts, respectively. In these fractions, phytosterols and triterpenes were analyzed after silanization, i.e., as volatile trimethylsilyl (TMS) derivatives, using GC-FID-MS (Table 3). In the case of unsaponifiable fraction of the roots extract (UFRE), phytosterols and triterpenes accounted for 93.7%, and the unsaponifiable fraction of the fruits extract (UFFE) accounted for 69.6% of the total unsaponifiable fractions. Among them, the dominant was *β*-sitosterol (60.0%) in UFRE, and a mixture of *β*-sitosterol and *α*-spinasterol (52.7%) in UFFE, followed by stigmasterol (18.5 and 26.3%) in both cases. A small quantity (4.1%) of one triterpene, *β*-amyrin, was detected in UFFE.

#### 2.1.4. Coumarin Composition of CH_2_Cl_2_ Extracts and Their MeOH Fractions (MFDEs)

Two coumarins, oxypeucedanin (**6**) and prantschimgin (**10**), were obtained as crystalline precipitates from the semi-solid (oily) CH_2_Cl_2_ extracts (**6** from the fruits extract and **10** from the roots extract) after treating the extracts with diethyl ether (precipitates accounted for 2.5 and 3.2% *w*/*w* of the fruits and roots extracts, respectively). The structures of these coumarins were elucidated by comparing ^1^H-NMR data with the literature [30,31,32]. The purity of the compounds was determined using LC-MS: **6** (78.20%) and **10** (93.55%).

**Figure 1:**
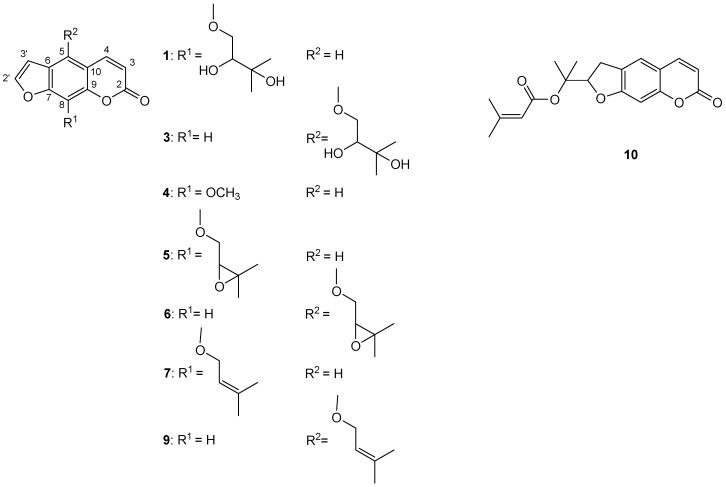
Structures of identified coumarins in MFDEs: **1**—heraclenol, **3**—oxypeucedanin hydrate, **4**—xanthotoxin, **5**—heraclenin, **6**—oxypeucedanin, **7**—imperatorin, **9**—isoimperatorin, **10**—prantschimgin.

To further investigate coumarins of *P. trifida* roots and fruits CH_2_Cl_2_ extracts, the MeOH fractions of CH_2_Cl_2_ extracts (MFDEs) were prepared; yields of MFDEs: 90.9% *w*/*w* (roots extract) and 69.5% *w*/*w* (fruits extract). The results are presented in Table 4 and Table 5 and Figure 1. LC–MS analysis of MFDEs enabled elucidation of the structures of ten coumarins [seven (**2**–**4**, **6**, **7**, **9**, **10**) in roots extract and nine (**1**–**3**, **5**–**10**) in fruits extract] (Table 4, Figure 1). The furanocoumarins xanthotoxin (**4**) and imperatorin (**7**) were identified using commercial standard compounds, and heraclenol (**1**) and heraclenin (**5**) using a previously chemically characterized CH_2_Cl_2_ extract of *Heracleum ternatum* Velen. fruits [33]. Based on comparison of their mass and UV spectra to those of imperatorin (**7**), heraclenol (**1**) and heraclenin (**5**), the next three furanocoumarins were identified as isoimperatorin (**9**), oxypeucedanin hydrate (**3**) and oxypeucedanin (**6**), respectively. Namely, isoimperatorin (**9**) had similar mass spectrum to imperatorin (**7**), oxypeucedanin hydrate (**3**) to heraclenol (**1**), and oxypeucedanin (**6**) to heraclenin (**5**). On the other hand, isoimperatorin (**9**), oxypeucedanin hydrate (**3**) and oxypeucedanin (**6**) had characteristic UV spectra of furanocoumarins oxygenated at C(5), in contrast with imperatorin (**7**), heraclenol (**1**) and heraclenin (**5**), whose UV spectra were typical for furanocoumarins oxygenated at C(8) [34,35]. This led to the conclusion that these three coumarin pairs [i.e., isoimperatorin (**9**) and imperatorin (**7**), oxypeucedanin hydrate (**3**) and heraclenol (**1**), and oxypeucedanin (**6**) and heraclenin (**5**)] are C(5)-C(8) positional isomers. The identity of oxypeucedanin (**6**) was confirmed using the external standard of this compound (obtained from *P. trifida* fruits crude CH_2_Cl_2_ extract). Compared to the identified furanocoumarins, compounds **2**, **8** and **10** had different UV spectra, similar to those of the simple coumarins. The coumarin **10** was identified as prantschimgin (senecioyl ester of marmesin) using the external standard of this 2′,3′-dihydrofuranocoumarin (obtained from *P. trifida* roots crude CH_2_Cl_2_ extract). The compound **8** had the same UV and mass spectra as **10**, suggesting that this was also a 2′,3′-dihydrofuranocoumarin derivative. The compound **2** had the same UV, but a different mass spectrum, compared to **10**, which indicated that this is a 2′,3′-dihydrofuranocoumarin without senecioyl group, most likely marmesin, a precursor in the biosynthesis of all abovementioned coumarins [36].

Quantitative LC–MS analysis of the MFDEs was performed using the external standard method (Table 5). Besides in the analyzed MFDEs, the contents of coumarins in the crude (whole) CH_2_Cl_2_ extracts were calculated. The dominant coumarin in the root isolates was prantschimgin (**10**; 118.7 mg/g MFDE and 107.9 mg/g extract), followed by imperatorin (**7**; 36.6 mg/g MFDE and 33.2 mg/g extract). In the fruit isolates, oxypeucedanin (**6**; 346.0 mg/g MFDE and 240.4 mg/g extract), prantschimgin (**10**; 96.3 mg/g MFDE and 66.9 mg/g extract) and oxypeucedanin hydrate (**3**; 87.5 mg/g MFDE and 60.8 mg/g extract) prevailed.

### 2.2. Antimicrobial and Antibiofilm Activities of P. trifida

#### 2.2.1. Antimicrobial Activity of Essential Oils

Antimicrobial activity of the essential oils of *P. trifida* roots, leaves, stems and fruits was tested against 13 microorganisms (Table 6). It was revealed that all investigated essential oils showed considerable antifungal activity against all six tested strains of molds from *Aspergillus*, *Penicillium* and *Trichoderma* genera (MIC = 0.10–0.78 mg/mL; MFC = 0.20–1.56 mg/mL), whereas the effect on *Candida albicans* was somewhat lower (MIC = 1.56–3.13 mg/mL; MFC = 3.13–6.25 mg/mL). The highest antifungal activity was demonstrated for all investigated essential oils against both tested *Penicillium* strains, the root and leaf oils against *T. harzianum*, and the root oil against *A. fumigatus* (MIC = 0.10 mg/mL; MFC = 0.20 mg/mL), followed by the stem and fruit oils against *A. fumigatus*, root and stem oils against *A. niger*, and stem oil against *T. harzianum* (MIC = 0.20 mg/mL; MFC = 0.39 mg/mL). Every investigated essential oil also exhibited activity on all six tested bacteria (MIC = 0.20–6.25 mg/mL; MBC = 0.39–12.5 mg/mL). The highest antibacterial effect was observed for the root and stem oils against *Staphylococcus aureus* and *Bacillus cereus* (MIC = 0.20 mg/mL; MBC = 0.39 mg/mL).

#### 2.2.2. Antibiofilm Activity of Essential Oils

Antibiofilm activity of the essential oils of *P. trifida* roots, leaves, stems and fruits was assessed against bacterial *Staphylococcus aureus*, *Listeria monocytogenes* and *Escherichia coli*, and fungal *Candida albicans* biofilms (Table 7). All tested essential oils exhibited a noteworthy inhibition of the formation of all investigated bacterial biofilms. The most intriguing observation within our study pertained to the significant inhibition of *L. monocytogenes* biofilm formation, with root and leaf essential oils demonstrating reductions of 80.2% and 82.6%, respectively. These effects were achieved when the oils were applied at sub-inhibitory concentrations, specifically at half of their minimum inhibitory concentrations (½MIC). Moreover, the leaf essential oil achieved high inhibition (50.3%) of the biofilm formation by this pathogen even in a lower concentration (¼MIC). Another noteworthy result pointed to the inhibition of *E. coli* biofilm formation by the fruit and root essential oils (46.3% and 27.3%); in both instances, the oils were applied in sub-inhibitory concentrations (½MIC). Finally, the root and stem essential oils that were applied at concentrations equal to half of their minimum inhibitory concentrations (½MIC), which is particularly noteworthy given their low MIC value of 0.2 mg/mL, exhibited effectiveness in inhibiting the formation of *S. aureus* biofilm, resulting in reductions of 37.7% and 35.5%, respectively. In contrast to tested bacterial biofilms, *C. albicans* biofilm was not significantly affected by any tested essential oil.

#### 2.2.3. Antimicrobial Activity of MFDEs

The experiments performed on *P. trifida* roots and fruits MFDEs, using the same 13 microorganisms (like in investigations of essential oils), provided intriguing results, both in terms of antibacterial (MIC = 0.125–4 mg/mL; MBC = 0.25–8 mg/mL) and antifungal activities (MIC = 0.0625–1 mg/mL; MFC = 0.125–2 mg/mL) (Table 8).

When it comes to antibacterial effects, *Bacillus cereus* emerged as the most sensitive strain in relation to the action of these MFDEs. This pathogen was particularly susceptible to the impacts of the MFDEs, exhibiting an MIC value of 0.125 mg/mL and an MBC value of 0.25 mg/mL. This indicates that *B. cereus* was affected by the smallest concentration of the MFDEs among all the tested bacterial strains, demonstrating its high sensitivity to the MFDEs.

As for antifungal activity, it was observed that the MFDEs displayed identical effects on each tested fungal species. The fungal species *Aspergillus niger* was found to be the most susceptible to the antifungal properties of the MFDEs, with an MIC value of 0.0625 mg/mL and an MFC value of 0.125 mg/mL. Notable activities of both MFDEs were also shown against *A. fumigatus* (MIC = 0.25 mg/mL; MFC = 0.5 mg/mL), whereas effects against *A. versicolor* and *Penicillium funiculosum* were somewhat lower (MIC = 0.5 mg/mL; MFC = 1 mg/mL).

#### 2.2.4. Antibiofilm Activity of MFDEs

Our study also explored the *P. trifida* roots and fruits MFDEs’ effectiveness in impeding the formation of *Staphylococcus aureus*, *Listeria monocytogenes*, *Escherichia coli* and *Candida albicans* biofilms (Table 9).

The most considerable inhibition of bacterial biofilm formation was noted in the case of *S. aureus*, when the fruits MFDE was applied at a sub-inhibitory concentration, which is half of the minimum inhibitory concentration (½MIC). Under this condition, the fruits MFDE was able to inhibit *S. aureus* biofilm formation by 28.3%. In a similar vein, the roots MFDE demonstrated appreciable *L. monocytogenes* biofilm inhibition when applied at one-fourth of the minimum inhibitory concentration (¼MIC). Under this condition, it was capable of inhibiting biofilm formation of this pathogen by 27.6%. On the other hand, neither MFDE inhibited formation of *E. coli* biofilm to a notable extent.

Our results also suggest that both MFDEs hold substantial promise in inhibiting *C. albicans* biofilm formation. Namely, antibiofilm activity was noteworthy in the case of almost all applied concentrations of the roots and fruits MFDEs, reaching 53.5 and 48.6% (at MICs), respectively.

#### 2.2.5. Testing MFDEs in Congo Red and Ergosterol Binding Assays in *Candida albicans*

Tested *P. trifida* roots and fruits MFDEs caused marked reduction in exopolysaccharide (EPS) production in *C. albicans* in congo red binding assay (Table 10). Specifically, when *C. albicans* was exposed to the fruits MFDE at its MIC, we noted a 31.5% reduction in EPS production. This reduction was even more pronounced (33.7%) when the roots MFDE was applied. Moreover, this effect was observed at ¼MIC.

The results of ergosterol binding assay were negative in the case of both MFDEs, i.e., no changes in the MIC values were observed after the addition of exogenous ergosterol.

## 3. Discussion

### 3.1. Chemical Composition of P. trifida

#### 3.1.1. Composition of Volatile Constituents

In this work, the chemical composition of *P. trifida* root, leaf, stem and fruit essential oils isolated by hydrodistillation was investigated. Obtained results were additionally confirmed by analyzing the composition of the headspace volatiles of these plant organs. Namely, static headspace extraction is a nondestructive, solvent-free and rapid method for the isolation of the most volatile components from plants [37]. Thus, using this procedure and subsequent GC-FID-MS analysis, the presence of the most volatile constituents of *P. trifida* essential oils was also revealed in the intact dried plant material. It should be noted that headspace volatiles are obtained in a gaseous state and cannot be used in investigations of antimicrobial and antibiofilm activities.

Previously, the essential oils of *P. trifida* originating from some other regions were analyzed. In comparison with previously investigated fruit essential oils, the sample from Serbia, investigated in the current work, was similar to those from Spain and Crimea and different from the one from Turkey [23,24,25]. Namely, the fruit oils from two localities in Spain and one in Crimea were also dominated by *p*-cimene (14.1, 24.5 and 9.1%), limonene (14.2, 34.5 and 31.3%) and *γ*-terpinene (28.3, 37.3 and 12.5%) [24,25]. On the other hand, in the fruit oil from Turkey the sesquiterpene *α*-humulene (33.1%) was the most abundant and it was followed by *p*-cymene (9.3%) [23]. *α*-Humulene was present in very low amounts in the samples from Serbia (2.1%) and Spain (traces), and it was absent in the sample from Crimea [24,25]. Regarding previously investigated root essential oils from two localities in Spain, they had similar compositions to the Serbian root essential oil sample: terpinolene (61.8 and 70.7%), *γ*-terpinene (8.2 and 13.6%) and *p*-cymene (8.0 and 10.9%) prevailed [24]. In the case of Spanish plants, essential oils of leaves with stems from four localities were also investigated. In the oils from two localities, (*E*)-*β*-ocimene was dominant (61.0 and 70.7%), as was also in the case in leaf essential oil of the plant from Serbia, while in the oils from the other two localities, the isomer (*Z*)-*β*-ocimene prevailed (20.5 and 51.5%) [24]. This isomer was also the dominant in the aerial parts (collected before flowering) essential oil of Italian *P. trifida* (18.1%) [26,27], while it was present in low quantities in the leaf and stem essential oils of the plant from Serbia (≤1.3%).

Regarding essential oils of other *Prangos* species, monoterpene hydrocarbons, such as α-pinene, *β*-pinene, *γ*-terpinene, *β*-phellandrene and *p*-cymene, were usually the most abundant [6]. As expected, some of them were also present as major and/or minor constituents in the *P. trifida* essential oils investigated in our work. However, one of specificities of investigated *P. trifida* essential oils was the dominance of *p*-cymen-8-ol in the stem essential oil (21.8%) and its presence in notable amounts in the root and leaf essential oils (6.2 and 13.0%). In the essential oils of other *Prangos* species, this oxygenated monoterpene was usually present in lower amounts or it was absent [6].

Our study is the first to reveal the chemical composition of essential oils isolated from *P. trifida* roots, leaves, stems and fruits collected in Serbia. Moreover, through comparison with previously published data [23,24,25,26,27], we provided initial insights about variation in the composition of *P. trifida* essential oils depending on the geographical origin of this species, and established a good basis for further research on this topic.

#### 3.1.2. Chemical Composition of CH_2_Cl_2_ Extracts

In *P. trifida* roots and fruits CH_2_Cl_2_ extracts, fatty acids, phytosterols, triterpenes and coumarins were investigated. Fatty acids, phytosterols and triterpenes were analyzed by GC-FID-MS. In that aim, more volatile derivatives of these compounds were prepared: methyl esters of fatty acids (FAME) and trimethylsilyl (TMS) derivatives of phytosterols and triterpenes. Regarding coumarins, two of them were obtained from CH_2_Cl_2_ extracts and identified using ^1^H-NMR. In order to further investigate these secondary metabolites, i.e., to perform their LC–MS analysis, MeOH fractions of CH_2_Cl_2_ extracts (MFDEs) were prepared. Namely, semi-solid (oily) CH_2_Cl_2_ extracts cannot be directly injected into a standard reversed-phase LC–MS system and coumarins have very similar solubility in CH_2_Cl_2_ and MeOH, i.e., those extracted using CH_2_Cl_2_ from the plant material will be also dissolved by MeOH [38].

In comparison with the current study, a very similar fruits fatty acid pattern, i.e., the prevalence of petroselinic acid (47.1–56.9%), which was followed by linoleic (21.8–30.4%) and oleic acids (9.5–14.0%), was observed for five Turkish *Prangos* species (*P. meliocarpoides*, *P. pabularia*, *P. platychlaena*, *P. uechtritzii* and *P. uloptera* DC.) [39,40]. On the other hand, the fatty acids in roots of *Prangos* species were investigated for the first time in our study. The abundance of petroselinic acid in *P. trifida* fruits extract and its absence from the roots extract, in which widespread linoleic acid was present in markedly higher amounts compared to other compounds, is in accordance with literature data for other Apiaceae species [39].

The two ubiquitous phytosterols *β*-sitosterol and stigmasterol, which were dominant in unsaponifiable fractions of both roots and fruits extracts (UFRE and UFFE) investigated in our work, were also previously isolated from the roots of *P. hulusii* [13]. Up to date, there is no literature data on phytosterols and triterpenes from the fruits of *Prangos* species. For example, *α*-spinasterol, which was also present in notable amounts in UFFE, was previously identified in some spices originating from the Apiaceae family, such as fruits of anise (*Pimpinella anisum* L.) and ajwain (*Trachyspermum ammi* L.) [41].

Regarding coumarins, it should be noted that prantschimgin (**10**), imperatorin (**7**) and isoimperatorin (**9**), identified in both roots and fruits (i.e., in their MFDEs) in the current work, were also previously identified in *P. trifida*, but the plant part from which these coumarins were isolated was not reported in the available literature [28]. These and all other coumarins identified in our study were also previously identified in different plant parts of various *Prangos* species [6]. For example, heraclenol (**1**), oxypeucedanin hydrate (**3**), heraclenin (**5**), oxypeucedanin (**6**), imperatorin (**7**), isoimperatorin (**9**) and prantschimgin (**10**) were isolated from the chloroform extract of the roots of *P. pabularia* [12]. Also, heraclenin (**5**) was identified in *P. heyniae* aerial parts MeOH and water extracts, and imperatorin (**7**) in *P. meliocarpoides* aerial parts hexane, ethyl acetate, MeOH and water extracts. Quantitative analysis of these extracts revealed that amounts of coumarins in the aerial parts of these species (phenophase was not specified) were much lower (up to 14.72 mg/g in the case of **7** in *P. meliocarpoides* water extract) [15] compared to *P. trifida* roots and fruits investigated in the current work. Similarly, in MeOH fractions of CH_2_Cl_2_ extracts (MFDEs) of *P. trifida* leaves and stems, prepared in the same way as MFDEs of roots and fruits in this work, we established notably lower amounts of coumarins (only up to 5 mg/g) using LC–MS. Therefore, as well as because fatty oils are present only in roots and fruits of this plant, CH_2_Cl_2_ extracts and MFDEs of leaves and stems were not the focus of this work.

Prantschimgin (**10**), which was firstly isolated from the ethanol extract of the roots of *P. tschimganica* O. Fedtsch. [42], is certainly the most specific coumarin identified in our work. Besides in the *Prangos* species, this coumarin was reported only for representatives of a few other related Apiaceae genera, e.g., *Ferulago* W.D.J.Koch [14].

In summary, the present study is, to the best of our knowledge, the first investigation of fatty acid, phytosterol and triterpene profiles of *P. trifida*. It also notably complements the data on coumarin composition of this plant, as it led to the identification of five new coumarins for this species.

### 3.2. Antimicrobial and Antibiofilm Activities, and Potential Mechanisms of Selected P. trifida Isolated Products

In our study, we adopted a multifaceted approach to evaluate the antimicrobial and antibiofilm properties of selected isolates derived from *P. trifida*. Specifically, we chose to investigate essential oils and coumarin-rich MeOH fractions of CH_2_Cl_2_ extracts (MFDEs). Our decision to focus on these particular isolates was guided by the presence and amounts of metabolites that we identified during our phytochemical investigations, as described in Section 2.1 (MFDEs contained notably higher shares of coumarins than crude CH_2_Cl_2_ extracts; Table 5). By focusing on these key antimicrobial constituents, we gained a deeper understanding of the potential antimicrobial properties of *P. trifida* isolates. In doing so, we contributed to a more thorough understanding of the biological properties of *P. trifida* isolates, shedding light on their potential applications in antimicrobial or antibiofilm contexts.

For this research, we selected 13 microorganisms, most of which are generally known to be food contaminants. They can cause food spoilage, produce various harmful toxins and/or cause foodborne infections. However, it should be noted that a number of tested microorganisms can also be spread in various other ways. Among these 13 microorganisms, 4 were selected for antibiofilm activity study. Due to biofilm production, they are significant from the aspect of food safety and/or hospital-acquired infections [43,44,45,46,47,48].

#### 3.2.1. Antimicrobial and Antibiofilm Activities of Essential Oils

There is an ongoing trend in the search for essential oils that can be used as new natural food preservatives. Moreover, essential oils are also being investigated as potential new natural products for the treatment of infections. Thus, we compared antimicrobial activity revealed for investigated essential oils of *P. trifida* roots, leaves, stems and fruits to the activity of the two commercial food preservatives of synthetic origin (sodium benzoate—E211 and potassium metabisulphite—E224), as well as to the activity of the standard antibiotic streptomycin and antimycotic ketoconazole. The most intriguing were the effects of all investigated essential oils against all six tested strains of molds, as well as of the root and stem oils against *Staphylococcus aureus* and *Bacillus cereus*, since they were all better compared to the effects of both E211 and E224 against these microorganisms. Despite essential oils generally showing weaker activity compared to standard drugs, it should be noted that obtained MIC values were in many cases ca. 0.1 mg/mL, which is regarded, according to some authors [49], as promising and justifies deeper investigations. Such MIC values were exhibited by all investigated essential oils against both tested *Penicillium* strains, the root, leaf and stem oils against *Trichoderma harzianum*, the root, stem and fruit oils against *Aspergillus fumigatus*, and the root and stem oils against *A. niger*, *S. aureus* and *B. cereus.* Generally, tested essential oils showed good potential for control of tested *Aspergillus* and *Penicillium* species, which are known food contaminants and producers of a number of potentially carcinogenic mycotoxins. For example, *A. niger* and *P. verrucosum* produce nephrotoxic, hepatotoxic, teratogenic and immunosuppressive ochratoxin A [43,44].

Furthermore, all investigated *P. trifida* essential oils (i.e., those of roots, leaves, stems and fruits) strongly inhibited biofilm formation by all tested bacteria (*Listeria monocytogenes*, *Escherichia coli* and *S. aureus*), but not of the tested yeast (*Candida albicans*). The significance of these results is reflected in the fact that *L. monocytogenes*, *E. coli* and *S. aureus* biofilms are emerging as a major safety concern in food processing plants due to their persistence on various surfaces, causing food spoilage and even foodborne diseases. Furthermore, *E. coli* and *S. aureus* biofilms are, among others, also found on medical devices in healthcare facilities and can cause hospital-acquired infections [45,46,47,48].

In previous studies [50,51,52,53,54], both the antimicrobial and antibiofilm activities against some microorganisms tested in our work, such as *S. aureus*, *L. monocytogenes* or *E. coli*, were demonstrated for certain essential oil constituents. For example, limonene, *α*-pinene, *p*-cymene, *γ*-terpinene and (*E*)-caryophyllene, which were present as either major or minor components in investigated *P. trifida* essential oils, exhibited these activities [50,51,52,53,54]. This fact can at least partly explain the activities observed in the current study.

Our study significantly widens the knowledge of antibacterial activity of *P. trifida* essential oils, which has been hinted at in previous research in which the authors investigated the potential of essential oil of aerial parts of this plant from Italy (collected before flowering) against six bacteria. The activity of the Italian essential oil was the same as the activity of the root and stem oils, investigated in the current work, against *B. cereus* (MIC = 0.2 mg/mL). The sample from Italy was also effective towards *B. subtilis* (MIC = 0.12 mg/mL). Activity was not found in the case of *S. aureus*, *E. coli*, *Pseudomonas aeruginosa* and *Salmonella* Typhimurium. However, it should be noted that concentrations of the oil of only up to 0.2 mg/mL were tested [26]. Regarding other *Prangos* species, antibacterial and/or antifungal activities were for example previously demonstrated for essential oils of *P. ferulacea* roots, leaves and flowers, *P. peucedanifolia* leaves and fruits, and *P. pabularia*, *P. asperula*, *P. platychlaena* and *P. uechtritzii* fruits [6,8,55,56,57,58,59]. However, antibiofilm activity of *Prangos* essential oils was not previously studied.

#### 3.2.2. Antimicrobial and Antibiofilm Activities of MFDEs

Coumarin-rich *P. trifida* roots and fruits MFDEs were investigated in a search for sources of compounds that could potentially represent new therapeutic options against the 13 aforementioned tested microorganisms. Thus, obtained MIC and MBC/MFC values were compared to those of standard antibiotic streptomycin and antimycotic ketoconazole. Despite the fact that MFDEs were active against all 13 microorganisms, the antimicrobial effects of the standard drugs were generally better. However, several notable results were revealed.

Overall, it is noteworthy that the antifungal impact of the tested MFDEs appeared to be more pronounced, or at least more evident than the antibacterial effect. Namely, in the case of *Penicillium funiculosum* and three tested *Aspergillus species*, MICs were below 1 mg/mL, and in the case of *A. niger*, even below 0.1 mg/mL. In addition to being producers of mycotoxins, some of these molds can cause serious infections in immunocompromised people, such as invasive aspergillosis caused by *A. fumigatus* [60]. Regarding tested bacteria, foodborne pathogen *Bacillus cereus* was by far the most susceptible microorganism in the case of both MFDEs with MICs close to 0.1 mg/mL.

Amongst coumarins identified in investigated MFDEs, antimicrobial activity was previously shown for prantschimgin (**10**) against *Staphylococcus aureus*, *Escherichia coli* and *Candida albicans* [61], imperatorin (**7**) against *E. coli* and *C. albicans* [62], as well as for oxypeucedanin (**6**), oxypeucedanin hydrate (**3**), and isoimperatorin (**9**) against all bacteria tested in the present study (including *B. cereus*) [63], at least partly clarifying our results. Regarding coumarin-rich isolated products of other *Prangos* species, for the CH_2_Cl_2_ extract of the roots of *P. hulusii*, as well as for its ten isolated coumarins (including **6** and **9**), the activity against 15 bacterial strains, including *S. aureus* and *E. coli* strains, was shown [13]. Very interesting effects of MFDEs tested in our work against molds, particularly *A. niger*, demand further research in order to reveal their active constituents.

The best biofilm inhibiting abilities of both the roots and fruits MFDEs were revealed in the case of *C. albicans*. Biofilms are an important aspect of this yeast’s life cycle and contribute to its ability to resist antimicrobial treatments and cause infections (e.g., oral thrush and vaginitis) [64]. Therefore, the potential of these MFDEs to reduce biofilm formation by about 50% at their MICs indicates that they may be useful tools in the fight against *C. albicans* infections. By inhibiting the biofilm formation of *C. albicans*, these MFDEs could hinder the yeast’s ability to establish resilient communities, thereby potentially facilitating its control and elimination. This is particularly important because biofilms are often linked to the development of chronic and recurrent infections that are difficult to treat with traditional antifungal agents. Besides on formation of *C. albicans* biofilm, the fruits MFDE also had notable effect on formation of *S. aureus* biofilm and the roots MFDE on formation of *Listeria monocytogenes* biofilm. Previously, reduction in the transcription of genes involved in the biofilm formation ability of *L. monocytogenes* was shown for *P. ferulacea* water extract [17].

However, despite the aforementioned biofilm inhibition capabilities, it is crucial to note that the efficiency of these MFDEs was not universal across all tested microorganisms. For example, these MFDEs exhibited a markedly reduced effect on the biofilm formation of the bacterium *E. coli*. In the case of this bacterium, the biofilm inhibitory effect of both MFDEs was minimal, showing that *E. coli* was relatively resilient to the biofilm inhibition properties of the MFDEs. This outcome suggests that the biofilm formation of *E. coli* might be governed by more complex mechanisms that may not be easily disrupted by these MFDEs, and warrants further investigation to understand the underlying processes. Our findings highlight the significant biofilm inhibition capability of the MFDEs against *C. albicans* when applied at their MICs. This provides a promising foundation for future research, possibly leading to the development of novel treatment strategies to combat infections caused by *C. albicans*.

In addition, the demonstrated antimicrobial activity of these extracts’ fractions represent a good basis for future investigation of the possibility of their application for the synthesis of bioactive multicomponent nanoparticles, in accordance with the growing trend in this field [65,66].

It should be noted that despite there are previous studies about the antimicrobial activity of coumarins identified in investigated MFDEs, data on their antibiofilm activity are lacking, which justifies further research on this matter.

#### 3.2.3. Potential Mechanisms of Action against Candida albicans of MFDEs

Our study explored potential mechanisms of action through which the MFDEs might inhibit *C. albicans*, a common fungal pathogen known for its ability to form biofilms that contribute to its resistance against antifungal treatments. The MFDEs were selected for testing their possible mechanisms because their MIC values were lower compared to the MIC values of the studied essential oils.

One significant observation from our results was the marked reduction in exopolysaccharide (EPS) production in *C. albicans*. EPS, a crucial component of the biofilm matrix that provides a protective barrier for biofilm-forming microorganisms, was noticeably reduced in the presence of both roots and fruits MFDEs. This substantial decrease in EPS production implies that these extracts may interfere with the EPS synthesis or export process, disrupting the formation of the protective biofilm matrix and leaving the fungal cells more vulnerable.

However, when we explored whether the antimicrobial action of the MFDEs could be attributed to binding to ergosterol—a major component of the fungal cell membrane—we found no changes in the MICs using the ergosterol binding assay. Ergosterol plays a vital role in maintaining the integrity, fluidity, and functionality of the fungal cell membrane, and many antifungal drugs exert their effects by interacting with ergosterol or inhibiting its synthesis [67]. However, the lack of change in MICs in our experiment indicates that the antimicrobial action of these MFDEs is not due to an interaction with ergosterol. Therefore, binding to ergosterol does not seem to be a mechanism of antimicrobial action for these MFDEs.

While the exact mechanisms of action remain to be fully elucidated, our study has revealed valuable insights into how these MFDEs might impede biofilm formation and disrupt the growth of *C. albicans*. The substantial reduction in EPS production is a promising lead for further investigation into the potential applications of these MFDEs in the fight against *C. albicans* and other biofilm-forming pathogens.

## 4. Materials and Methods

### 4.1. Plant Material

The plant material (roots, leaves, stems and fruits of *P. trifida*) was collected in Sićevo Gorge (Kusača), Serbia in 2020 (22.0810369° E, 43.315107° N). The plant was identified by Dr. Marjan Niketić, curator/botanist of the Natural History Museum, Belgrade (Serbia), and the voucher specimen is deposited in the Herbarium of the Natural History Museum, Belgrade (BEO); voucher number: 20200603. The plant material was air-dried prior to analyses.

### 4.2. Isolation of the Essential Oils

The powdered roots, leaves, stems and fruits were hydrodistilled for 2.5 h using a Clevenger-type apparatus, according to the procedure of European Pharmacopoeia 11.0 [68]. *n*-Hexane was used as the collecting solvent. Essential oils were dried over anhydrous sodium sulfate, *n*-hexane was evaporated, and essential oils were stored at 4 °C until analysis. The determination of essential oil content was done in triplicate.

### 4.3. Chemical Analysis of the Essential Oils

GC-FID-MS analysis was performed on an Agilent 6890N Gas Chromatograph (Agilent Technologies, Palo Alto, CA, USA), equipped with split/splitless injector, capillary column (Agilent HP-5MS 30 m × 0.25 mm, 0.25 μm film thickness) and flame ionization detector (FID), and coupled to an Agilent 5975C MS detector [33]. Briefly, injector and FID temperatures: 200 and 300 °C, respectively. Carrier gas: helium; flow: 1.0 mL/min. The oven temperature: 60 to 280 °C at 3 °C/min (final temperature held for 10 min). Split ratio: 1:10. Injected volume: 1 μL of 1.5% (*v*/*v*) solutions of essential oils in *n*-hexane. MSD was operating in EI mode at 70 eV. MSD transfer line, ion source and analyzer (single quadrupole) temperatures: 250, 230 and 150 °C, respectively. Range *m/z*: 35–550. Scan speed: 2.83 scans/s. The analysis was done using the MSD ChemStation E.01.00.237 software. Linear retention indices (RIs) of the constituents were determined in relation to the homologue series of *n*-alkanes (C_8_-C_40_) (Fluka, Buchs, Switzerland) ran under the same operating conditions. The identification of the compounds was based on the comparison of their retention indices (RI) and mass spectra to those from the NIST/NBS 05 and Wiley 8th edition mass spectra libraries, as well as the literature [29]. Relative percentages of the compounds were calculated using peak areas from the FID data.

### 4.4. Static Headspace (HS) Extraction and Chemical Analysis of HS Volatiles

HS extractions were performed using an Agilent G1888 automatic HS sampler coupled with Agilent 6890N Gas Chromatograph. Roots, leaves, stems and fruits (0.5 g) were ground and hermetically sealed in HS vials. Experimental conditions of HS extractions were as follows [69]: oven temperature 90 °C, loop temperature 100 °C, transfer line 110 °C, equilibration time 30 min, shaking low; pressurization time 0.08, carrier gas helium, in vial pressure 15 psi, loop fill 0.5, loop equilibration 0.05, inject time 1.00. GC-FID-MS experimental conditions, as well as methods of identification and quantification of compounds, were the same as in the case of the essential oils analysis.

### 4.5. Obtaining of CH_2_Cl_2_ Extracts

Roots and fruits were powdered and extracted with CH_2_Cl_2_ at room temperature. Two successive extractions with CH_2_Cl_2_ were performed. The first extraction lasted for 3 days, and then after filtration, the residual material was subjected to the second extraction, which lasted for 2 days (herbal drug:solvent ratio in both steps of extraction 1:10 *w*/*v*). After filtration, two resulting extracts were combined. CH_2_Cl_2_ was removed under reduced pressure to obtain semi-solid (oily) extracts.

### 4.6. Saponification and Transesterification of CH_2_Cl_2_ Extracts

The saponification of the fatty oils in 1 g of roots and fruits CH_2_Cl_2_ extracts was done using 50% potassium hydroxide (5 mL)/ethanol (30 mL) at 90 °C for 60 min. Unsaponifiable fractions were separated using petroleum ether, and the soap-rich polar fractions were treated with hydrochloric acid to obtain free fatty acids, which were then collected using diethyl ether. Afterwards, the fatty acids were esterified using 98% sulphuric acid (1 mL)/methanol (150 mL, purity ≥ 99.9%) at 80 °C for 60 min to obtain volatile fatty acid methyl esters (FAME), which were then collected using petroleum ether. To analyze phytosterols and triterpenes, the unsaponifiable fractions (300 μL of 5 mg/mL solution in CH_2_Cl_2_) were treated with bis-(trimethylsilyl)-trifluoroacetamide (BSTFA; Sigma-Aldrich, St. Louis, MO, USA) (200 μL) and held at 60 °C for 60 min to obtain volatile trimethylsilyl (TMS) derivatives [70].

### 4.7. Analysis of the FAME, Phytosterols and Triterpenes in CH_2_Cl_2_ Extracts

The same GC-FID-MS system as in the case of the analysis of the essential oils was used, except that for analysis of FAME, different column (Agilent J&W HP-88 100 m × 0.25 mm, 0.20 μm film thickness) was installed. In the case of FAME, injector and FID temperatures were both 260 °C. Carrier gas: helium; flow: 1.2 mL/min. Oven temperature was initially held at 140 °C for 5 min, then increased linearly from 140 to 240 °C at 4 °C/min, and finally held at 240 °C for 10 min. Split ratio: 1:25. Injected volume: 1 μL of 1.0% (*v*/*v*) solution of FAME in CH_2_Cl_2_. MSD parameters were the same as for the analysis of essential oils. The identification of FAME was based on the comparison of their retention times (Rt) and mass spectra to those of commercial standards ran under the same chromatographic conditions (Supelco 37 Component FAME Mix, petroselinic acid methyl ester and *cis*-11-vaccenic acid methyl ester; all Sigma-Aldrich) [70].

The experimental conditions for the phytosterol and triterpene analysis were the same as in the case of the essential oils, except that for phytosterols and triterpenes, the final oven temperature (280 °C) was held for 20 min. The phytosterols and triterpenes were identified based on comparison of their mass spectra to those from the NIST/NBS 05 and Wiley 8th edition libraries, as well as the literature [41,71]. Identity of stigmasterol, *β*-sitosterol (both Val-de-Reuil, France) and *β*-amyrin (Sigma-Aldrich) was confirmed using commercial standard compounds.

The relative percentages of the FAME, phytosterols, and triterpenes were calculated based on the peak areas from the FID data.

### 4.8. Obtaining of Oxypeucedanin (***6***) and Prantschimgin (***10***) from CH_2_Cl_2_ Extracts

To semi-solid roots and fruits CH_2_Cl_2_ extracts (470.0 and 1000.0 mg, respectively), diethyl ether (5 mL in both cases) was added. Obtained solid (crystalline) precipitates were separated by filtering through filter papers and additionally washed with diethyl ether until oily parts were completely removed.

^1^H-NMR spectra of the crystalline precipitates were recorded on Avance III (Bruker, Billerica, MA, USA), operating at 400 MHz. CDCl_3_ (Sigma-Aldrich) was used as solvent and TMS as internal standard. The precipitate obtained from the fruits extract (25.0 mg) contained compound **6** and the one obtained from the roots extract (15.1 mg) contained compound **10**.

Oxypeucedanin (**6**) ^1^H-NMR (400 MHz, CDCl_3_, *J* in Hz): 8.21 (1H, d, *J* = 9.8, H-4), 7.62 (1H, d, *J* = 2.2, H-2′), 7.21 (1H, s, H-8), 6.95 (1H, d, *J* = 2.2, H-3′), 6.32 (1H, d, *J* = 9.8, H-3), 4.60 (1H, dd, *J* = 10.8, 4.3, H-1″), 4.44 (1H, dd, *J* = 10.8, 6.5, H-1″) 3.23 (1H, dd, *J* = 4.6, 6.2, H-2″), 1.41 (3H, s, Me-3″), 1.33 (3H, s, Me-3″).

Prantschimgin (**10**) ^1^H-NMR (400 MHz, CDCl_3_, *J* in Hz): 7.59 (1H, d, *J* = 9.5, H-4), 7.21 (1H, s, H-5), 6.74 (1H, s, H-8), 6.21 (1H, d, *J* = 9.5, H-3), 5.55 (1H, s, H-2 (OSen)), 5.14 (1H, t, *J* = 8.6, H-2′), 3.23 (2H, m, H-3′), 2.10 (3H, s, H-4 (OSen)), 1.85 (3H, s, H-5 (OSen)), 1.60 (3H, s, H-2″), 1.53 (3H, s, H-3″).

The purity of compounds was determined based on peak areas recorded on diode array detector (DAD) at 250 nm (**6**) and 350 nm (**10**), using the LC–MS method described in the Section 4.10. 

### 4.9. Obtaining of Methanol Fractions of CH_2_Cl_2_ Extracts (MFDEs)

The semi-solid roots and fruits CH_2_Cl_2_ extracts were suspended in MeOH (5 mg/mL) and filtered through membrane filters (0.45 μm). MeOH was removed from filtrate under reduced pressure.

### 4.10. LC–MS Analysis of Coumarins in MFDEs

The coumarins were analyzed on an Agilent Infinity 1200 Liquid Chromatograph (Agilent Technologies), equipped with autosampler, quaternary pump, Agilent Zorbax SB-Aq column (150 × 3.0 mm; 3.5 μm particle size), diode array detector (DAD) and Single quad MS detector with an electrospray ionization (ESI) ion source (LC–MS). MFDEs were dissolved in MeOH (5 mg/mL). Injection volume: 5 μL (roots MFDE) or 2.5 μL (fruits MFDE). Binary mobile phase, consisting of 0.1% formic acid and 10% isopropanol (A), and MeOH (B) (all Sigma-Aldrich; LC–MS purity), was applied at 0.3 mL/min; gradient: 25–100% B (30 min). DAD chromatograms were recorded at 210, 250, 270, 320 and 350 nm. ESI mass spectra were recorded in positive ion mode, with nebulization with nitrogen at 10 L/min and pressure of 40 psi, at temperature of 350 °C and capillary voltage at 3500 V. Signals were registered by fragmentor voltage of 100 V or 250 V. Analysis was done in triplicate.

Identification of coumarins was performed using commercial standards of xanthotoxin (**4**) and imperatorin (**7**) (obtained from Sigma-Aldrich), oxypeucedanin (**6**) and prantschimgin (**10**) obtained in this work, and previously chemically characterized CH_2_Cl_2_ extract of *Heracleum ternatum* fruits [33] (**1** and **5**), or their structures were elucidated to the highest possible extent based on analysis of their UV and mass spectra (**2**, **3**, **8** and **9**). Quantification of compounds was done using imperatorin (**7**), oxypeucedanin (**6**) and prantschimgin (**10**) by the external standard method using peak areas obtained by DAD at 250 nm (**7** and **6**) or 350 nm (**10**). Based on similarity of their UV spectra, imperatorin (**7**) was used for quantification of furanocoumarins oxygenated at C(8), oxypeucedanin (**6**) for furanocoumarins oxygenated at C(5), and prantschimgin (**10**) for 2′,3′-dihydrofuranocoumarins. For the purpose of quantification of isoimperatorin (**9**; which co-eluted with prantschimgin, **10**), the calibration curve of imperatorin (**7**) using peak area obtained by Single Ion Monitoring (SIM) of *m/z* 271.1 was used. Limits of detection (LODs) and quantification (LOQs) for the coumarins were determined using the standard deviations of the intercepts (SD_b_) and the slopes (a) as follows: LOD = 3.3 × SD_b_/a and LOQ = 10 × SD_b_/a. Regression equations, *r*^2^, LODs and LOQs are given in Table S1.

### 4.11. Investigation of Antibacterial and Antifungal Activities of Essential Oils and MFDEs

The following Gram-positive bacteria: *Staphylococcus aureus* (ATCC 11632), *Bacillus cereus* (food isolate) and *Listeria monocytogenes* (NCTC 7973), as well as Gram-negative bacteria *Escherichia coli* (ATCC 25922), *Enterobacter cloacae* (ATCC 35030) and *Salmonella* Typhimurium (ATCC 13311) were tested. Also, the following micromycetes were used: *Aspergillus fumigatus* (ATCC 9197), *Aspergillus niger* (ATCC 6275), *Aspergillus versicolor* (ATCC 11730), *Penicillium funiculosum* (ATCC 36839), *Penicillium verrucosum* var. *cyclopium* (food isolate), *Trichoderma harzianum* (TH-IS005-12) and *Candida albicans* (ATCC 10231). The microorganisms are deposited at Mycological laboratory, Department of Plant Physiology, Institute for Biological research “Sinisa Stanković”, National Institute of Republic of Serbia, University of Belgrade, Serbia. By using the microdilution method [72], minimal inhibitory concentrations (MICs) and minimal bactericidal/fungicidal concentrations (MBCs/MFCs) were calculated. The 96-well microtiter plates were incubated at 37 °C for 24 h with serially diluted essential oils/MFDEs in liquid broth. After incubation, the MICs and MFCs were identified. MIC was defined as the lowest concentrations at which no growth was seen under a microscope. After successive sub-cultivation of 10 µL of samples at 37 °C for 24 h, MBC/MFC values were observed as concentrations without discernible growth. As positive controls, artificial food preservatives E211 and E224, as well as standard antibiotic streptomycin (Sigma-Aldrich) and antimycotic ketoconazole (Sigma-Aldrich), were used.

### 4.12. Inhibition of Biofilm Formation of Essential Oils and MFDEs

Strains used for inhibition of biofilm formation assay were *Staphylococcus aureus* (ATCC 11632), *Listeria monocytogenes* (NCTC 7973), *Escherichia coli* (ATCC 25922) and *Candida albicans* (ATCC 10231).

The ability of essential oils/MFDEs to inhibit the formation of biofilm was examined as described previously [73]. Selected strains were cultured with MIC, ½MIC and ¼MIC concentrations of the essential oils/MFDEs in TSB/YPD medium at 37 °C for 24 h in 96-well microtiter plates with adhesive bottoms (Sarstedt, Germany). Following three sterile PBS (Phosphate Buffered Saline, pH 7.4) well washes, biofilms were fixed in methanol for 20 min. After that, the methanol was taken out and biofilms were dyed for 30 min with 0.1% crystal violet (Bio-Merieux, France). Ethanol (96%) (Zorka, Serbia) was applied to dissolve bonded crystal violet after the plate had been slowly cleaned (to remove excess dye) and dried on the air. Thermo Scientific’s Multiskan FC Microplate Photometer was used to measure the absorbance (620 nm). Percentage of inhibition of biofilm formation was calculated using absorbances of samples treated with essential oils/MFDEs (A_620_ sample) and untreated samples (A_620_ control), using the following equation:

Inhibition (%) = [(A_620_ control − A_620_ sample)/A_620_control)] × 100 (1)

### 4.13. Congo Red Binding Assay

According to a previously described approach [74], the effect of the tested MFDEs on the exopolysaccharide (EPS) synthesis by the *C. albicans* ATCC 10231 biofilm was assessed with some changes. Tested MFDEs were applied to preformed 24-h biofilms in microtiter plates for 24 h at 37 °C at its MIC, ½MIC and ¼MIC concentrations. The adherent cells were then rinsed with PBS after the planktonic cells were removed. Wells received congo red (1%, *w*/*v*) and were then left in the dark for 30 min. The excess dye was removed, and 200 μL DMSO was used to dissolve the bound congo red. A microtiter plate reader at 450 nm was used to measure the absorbance. Percentage of EPS inhibition was calculated using absorbances of samples treated with MFDEs (A_450_ sample) and untreated samples (A_450_ control), using the following equation:

Inhibition (%) = [(A_450_ control − A_450_ sample)/A_450_ control)] × 100 (2)

### 4.14. Ergosterol Binding Assay in C. albicans

Ergosterol binding assay was performed according to the method described by Leite et al. [64], with some modifications. Briefly, MFDEs microdilutions were prepared in a similar manner as for the testing of antimicrobial activity, with the exception that ergosterol (400 μg/mL) was added to the plate’s rows. After a 24-h incubation period at 37 °C, the MIC values of the samples without added ergosterol were contrasted with the MIC values of ergosterol-added samples. The increase of MIC value in ergosterol-added samples would indicate that the antimicrobial action is due to an interaction with ergosterol.

### 4.15. Statistical Analysis

The results were presented as the mean value of three replicates ± standard deviation (SD). When applicable, the data were analyzed by one-way analysis of variance (ANOVA) followed by Tukey’s HSD test with *α* = 0.05 using the program IBM SPSS version 22.0, IBM Corp., Armonk, NY, USA. Values of *p* < 0.05 were regarded for statistically significant.

## 5. Conclusions

In this work, the data about *P. trifida* volatile constituents and coumarins were significantly complemented, whereas fatty acids and phytosterols of this plant were, to the best of our knowledge, investigated for the first time. Essential oils showed strong activity against a wide range of food contaminants, as well as promising inhibition of biofilm formation of some tested bacteria, justifying their further investigation as raw materials for pharmaceutical and food industries. Also, results obtained for MFDEs provide a good basis for the investigation of antimicrobial and antibiofilm activities of identified coumarins, particularly against tested molds, as well as *Candida albicans.* As we move forward, continued exploration of *P. trifida* chemical composition and its practical applications in pharmaceuticals and food safety will undoubtedly pave the way for innovative solutions to real-world challenges in these industries.

## Figures and Tables

**Table 1:** Chemical composition of *P. trifida* volatile constituents.

RI_exp_ ^1^	RI_lit_ ^2^	Compound ^3^	Essential Oils, % ^4^				Headspace Volatiles, % ^4^			
			Root	Leaf	Stem	Fruit	Root	Leaf	Stem	Fruit
934	924	*α*-Thujene	-	-	-	-	tr	tr	tr	0.4
935	932	*α*-Pinene	1.3	11.3	5.7	7.0	5.0	17.3	21.7	19.5
950	946	Camphene	tr	0.5	0.9	tr	tr	0.6	1.1	tr
975	969	Sabinene	tr	0.4	tr	4.5	tr	0.5	tr	12.5
979	974	*β*-Pinene	-	0.4	tr	0.5	-	0.5	tr	-
992	988	Myrcene	-	1.0	tr	0.6	1.5	1.2	tr	1.2
992	984	2-Pentyl furan	2.6	-	tr	-	-	-	-	-
1004	998	*n*-Octanal	0.3	-	0.4	0.3	-	-	-	-
1007	1002	*α*-Phellandrene	tr	tr	-	0.8	tr	tr	-	1.7
1026	1020	*p*-Cymene	11.5	4.9	14.1	25.4	11.1	3.3	40.3	23.3
1030	1024	Limonene	4.7	6.2	3.4	14.4	11.6	6.9	21.6	23.9
1037	1032	(*Z*)-*β*-Ocimene	tr	1.3	tr	tr	-	3.0	-	-
1050	1044	(*E*)-*β*-Ocimene	tr	23.2	1.7	0.2	tr	40.9	2.5	-
1059	1054	*γ*-Terpinene	2.4	tr	tr	11.4	4.7	-	tr	15.4
1068	1065	*cis*-Sabinene hydrate	-	-	tr	0.2	-	-	-	-
1092	1086	Terpinolene	36.2	18.1	2.7	1.8	58.5	18.6	6.1	1.5
1092	1089	*p*-Cymenene	tr	tr	1.9	tr	-	-	-	-
1127	1122	*α*-Campholenal	tr	tr	1.5	tr	-	-	-	-
1140	1135	*trans*-Pinocarveol	-	tr	1.8	tr	-	-	-	-
1142	1137	*cis*-Verbenol	-	-	0.8	-	-	-	-	-
1142	1137	(*E*)-Epoxy-ocimene	-	0.6	-	-	-	0.2	-	-
1147	-	4,8-Epoxy-*p*-menth-1-ene	3.0	5.1	8.1	0.5	2.5	1.9	3.3	tr
1163	1160	Pinocarvone	-	-	0.6	tr	-	-	-	-
1178	-	1,8-Menthadien-4-ol	tr	1.2	6.6	0.9	-	-	-	-
1190	1179	*p*-Cymen-8-ol	6.2	13.0	21.8	1.3	5.3	1.5	3.4	tr
1199	1195	Myrtenal	-	tr	0.9	tr	-	-	-	-
1220	1215	*trans*-Carveol	-	tr	1.1	tr	-	-	-	-
1245	1239	Carvone	tr	tr	1.3	-	-	-	-	-
1286	1287	Bornyl acetate	0.9	tr	1.0	tr	-	-	-	-
1317	1315	(2*E*,4*E*)-Decadienal	1.1	-	tr	-	-	-	-	-
1357	1352	2,3,6-Trimethyl benzaldehyde	0.6	0.8	tr	tr	-	-	-	-
1392	1389	*β*-Elemene	2.6	tr	tr	4.2	tr	-	tr	0.3
1420	1417	(*E*)-Caryophyllene	0.7	1.2	1.9	1.2	tr	0.1	tr	tr
1434	1434	*γ*-Elemene	-	-	-	0.3	-	-	-	-
1455	1452	*α*-Humulene	tr	tr	tr	2.1	-	-	-	-
1482	1484	Germacrene D	-	-	-	0.5	-	-	-	-
1487	1489	*β*-Selinene	2.2	-	tr	0.6	-	-	-	-
1497	1498	*α*-Selinene	2.8	-	tr	-	-	-	-	-
1498	1498	*α*-Selinene + Bicyclogermacrene	-	-	-	1.4	-	-	-	-
1511	1505	*β*-Bisabolene	8.7	tr	tr	1.8	-	-	-	-
1543	1545	Selina-3,7(11)-diene	-	-	-	0.8	-	-	-	-
1561	1559	Germacrene B	-	-	-	5.6	-	-	-	-
1566	1561	(*E*)-Nerolidol	-	-	-	3.0	-	-	-	-
1581	1577	Spathulenol	-	-	-	1.2	-	-	-	-
1586	1582	Caryophyllene oxide	1.6	5.7	13.1	2.0	-	-	-	-
1611	1608	Humulene epoxide II	-	tr	0.7	0.7	-	-	-	-
1638	-	Caryophylla-dien-5-ol isomer	-	tr	1.2	tr	-	-	-	-
1656	1658	Selin-11-en-4-*α*-ol	0.9	-	tr	0.2	-	-	-	-
1844	-	Hexahydrofarnesyl acetone	-	0.3	0.6	tr	-	-	-	-
1866	-	Pentadecanoic acid	0.8	-	-	-	-	-	-	-
1971	1959	Hexadecanoic acid	3.8	-	-	-	-	-	-	-
2137	-	Octadecadienoic acid isomer	0.6	-	-	-	-	-	-	-
		Monoterpene hydrocarbons	56.2	67.2	30.5	66.5	92.3	93.0	93.3	99.4
		Oxygenated monoterpenes	10.0	19.9	45.6	2.9	7.7	3.6	6.7	tr
		Sesquiterpene hydrocarbons	17.0	1.2	1.9	18.4	tr	0.1	tr	0.3
		Oxygenated sesquiterpenes	2.5	6.0	15.6	7.1	-	-	-	-
		Others	9.9	0.8	0.4	0.3	-	-	-	-
		Total identified compounds	95.6	95.1	94.0	95.2	100.0	96.7	100.0	99.7
		No of identified compounds	32	32	39	43	15	16	15	14

^1^ RI_exp_, retention indices on HP-5MS column relative to C_8_-C_40_ *n*-alkanes. ^2^ RI_lit_, retention indices obtained from the literature [29]. ^3^ Constituents listed in the order of elution on HP-5MS column. ^4^ Relative area percentage of the compounds obtained from FID area percent data; tr, trace (<0.1%); -, not detected.

**Table 2:** Fatty acid composition of *P. trifida* roots and fruits CH_2_Cl_2_ extracts.

Rt ^1^	Fatty Acid ^2^	Roots Extract, % ^3^	Fruits Extract, % ^3^
15.03	Lauric acid (C12:0)	tr	-
18.30	Myristic acid (C14:0)	0.2	0.1
20.02	Pentadecanoic acid (C15:0)	1.5	0.1
21.75	Palmitic acid (C16:0)	14.9	5.6
22.83	Palmitoleic acid (C16:1n7c)	0.6	0.3
23.41	Heptadecanoic acid (C17:0)	0.4	0.1
25.04	Stearic acid (C18:0)	1.0	1.0
25.98	Petroselinic acid (C18:1n12c)	-	49.9
26.03	Oleic acid (C18:1n9c)	14.2	10.2
26.12	*cis*-Vaccenic acid (C18:1n7c)	1.9	1.0
27.37	Linoleic acid (C18:2n6c)	51.8	28.3
28.08	Arachidic acid (C20:0)	0.6	0.3
28.84	*α*-Linolenic acid (C18:3n3)	3.6	0.8
29.52	Heneicosanoic acid (C21:0)	tr	0.1
30.92	Behenic acid (C22:0)	0.7	0.4
32.33	Tricosanoic acid (C23:0)	0.3	0.2
33.80	Lignoceric acid (C24:0)	0.9	0.3
	Saturated fatty acids	20.4	8.2
	Monounsaturated fatty acids	16.8	61.3
	Polyunsaturated fatty acids	55.4	29.1
	Total identified fatty acids	92.6	98.7

^1^ Retention times on HP-88 column [min]. ^2^ Investigated as fatty acid methyl esters (FAME). ^3^ Relative percentages obtained from FID area percent data; tr, trace (<0.1%); -, not detected.

**Table 3:** Phytosterol and triterpene composition of unsaponifiable fractions of *P. trifida* roots and fruits CH_2_Cl_2_ extracts (UFRE and UFFE).

Rt ^1^	Compound ^2^	UFRE, % ^3^	UFFE, % ^3^
78.24	Campesterol	2.7	1.3
79.26	Stigmasterol	18.5	26.3
80.51	Δ^5,23^-Stigmastadienol	0.9	-
80.64	*β*-Amyrin	-	4.1
81.23	*β*-Sitosterol + *α*-spinasterol	-	52.7
81.29	*β*-Sitosterol	60.0	-
81.48	Stigmastanol	3.3	-
83.10	Δ^7^-Stigmastenol	2.8	7.1
83.71	Δ^7^-Avenasterol	1.1	1.7
	Total identified	89.4	93.3
	Total sterols/triterpenes in unsaponifiable fractions	93.7	69.6

^1^ Retention times on HP-5 column [min]. ^2^ Investigated as trimethylsilyl (TMS) derivatives. ^3^ Relative percentages obtained from FID area percent data.

**Table 4:** UV and MS data of coumarins identified in *P. trifida* roots and fruits MFDEs obtained by LC–MS in positive ESI mode at fragmentor voltage of 100 V and 250 V.

Rt, min	Coumarin (Registered Number) ^1^	*λ*_max_, nm	*m/z* (100 V)	*m/z* (250 V)
13.17	Heraclenol (**1**) ^2^	218, 248, 302	631.1 (36.5) ^4^, 327.0 (100.0), 322.1 (17.1), 287.0 (39.2), 269.10 (13.0), 121.2 (25.1)	327.1 (100.0), 240.9 (12.7), 203.0 (24.5), 173.0 (10.8), 147.10 (24.9), 129.0 (12.7)
13.81	2′,3′-Dihydrofuranocoumarin derivative (**2**)	222, 334	269.1 (33.5), 247.1 (100.0), 121.1 (16.6)	269.0 (100.0), 247.1 (51.5), 229.1 (40.1), 213.1 (12.6), 175.0 (58.3), 147.0 (31.6)
14.66	Oxypeucedanin hydrate (**3**)	222, 250, 266, 310	631.1 (100.0), 327.1 (12.2), 305.1 (94.8)	327.0 (100.0), 203.0 (51.3), 147.0 (18.1)
15.48	Xanthotoxin (**4**) ^3^	218, 248, 302	455.0 (49.3), 239.0 (22.4), 217.1 (100.0)	217.0 (13.5), 202.0 (72.1), 174.1 (100.0), 161.0 (15.6), 146.0 (13.5), 131.0 (12.4), 118.1 (14.8)
18.01	Heraclenin (**5**) ^2^	218, 248, 302	595.1 (84.1), 309.0 (81.8), 304.1 (15.2), 287.1 (100.0), 269.1 (70.5), 203.0 (10.6)	309.1 (100.0), 224.0 (15.7), 203 (61.7), 173.0 (24.7), 157.0 (16.7), 147.1 (63.9), 129.1 (29.4), 89.1 (19.6)
19.69	Oxypeucedanin (**6**)	222, 250, 266, 310	595.1 (64.5), 287.0 (100.0)	309.0 (100.0), 203.0 (57.4), 147.1 (37.1)
22.05	Imperatorin (**7**)	218, 248, 302	563.1 (100.0), 293.0 (33.9), 271.1 (87.9), 203.0 (43.0)	293.0 (12.3), 224.0 (43.3), 203.1 (35.6), 175.0 (11.5), 147.0 (100.0), 129.0 (26.3), 91.1 (13.7)
23.90	2′,3′-Dihydrofuranocoumarin derivative (**8**) ^2^	222, 334	679.2 (93.8), 351.1 (12.6), 329.1 (100.0)	679.2 (11.8), 351.1 (100.0), 229.1 (46.2)
24.29	Isoimperatorin (**9**)	222, 250, 266, 310	563.1 (100.0), 293.0 (11.8), 271.1 (93.3), 203.0 (33.7)	293.0 (100.0), 224.0 (16.1), 203.0 (35.9), 147.1 (86.9), 91.1 (28.5)
24.39	Prantschimgin (**10**)	222, 334	679.2 (75.4), 329.1 (100.0)	679.2 (11.5), 351.1 (100.0), 247.1 (11.9), 229.1 (40.2)

^1^ Compound number used in text; numbers are registered based on retention times (Rt), i.e., order of elution of compounds on SB-Aq column. ^2^ Detected only in the fruits MFDE. ^3^ Detected only in the roots MFDE. ^4^ Numbers in brackets refer to the relative abundances of the ions [%].

**Table 5:** Coumarin composition of *P. trifida* roots and fruits MFDEs.

Rt, min	Coumarin (Registered Number) ^1^	Content in MFDEs and Expressed on the Crude (Whole) CH_2_Cl_2_ Extracts, mg/g			
		Root MFDE	Root Extract	Fruit MFDE	Fruit Extract
13.17	Heraclenol (**1**) ^2^	-	-	4.1 ± 1.8	2.8 ± 1.2
13.81	2′,3′-Dihydrofuranocoumarin derivative (**2**) ^3^	3.1 ± 0.2	2.8 ± 0.1	6.8 ± 0.6	4.7 ± 0.4
14.66	Oxypeucedanin hydrate (**3**) ^4^	22.4 ± 0.1	20.3 ± 0.1	87.5 ± 5.9	60.8 ± 4.1
15.48	Xanthotoxin (**4**) ^2^	11.0 ± 0.9	10.0 ± 0.8	-	-
18.01	Heraclenin (**5**) ^2^	-	-	33.8 ± 10.3	23.5 ± 7.2
19.69	Oxypeucedanin (**6**)	18.4 ± 3.4	16.7 ± 3.1	346.0 ± 33.7	240.4 ± 23.4
22.05	Imperatorin (**7**)	36.6 ± 2.5	33.2 ± 2.3	32.5 ± 4.2	22.6 ± 2.9
23.90	2′,3′-Dihydrofuranocoumarin derivative (**8**) ^3^	-	-	54.6 ± 0.0	37.9 ± 0.0
24.29	Isoimperatorin (**9**) ^2^	9.4 ± 0.9	8.5 ± 0.8	28.3 ± 8.3	19.7 ± 5.8
24.39	Prantschimgin (**10**)	118.7 ± 0.1	107.9 ± 0,1	96.3 ± 1.1	66.9 ± 0.7

^1^ Compound number used in text; numbers are registered based on retention times (Rt), i.e., order of elution of compounds on SB-Aq column. ^2^ Calculated as imperatorin. ^3^ Calculated as prantschimgin. ^4^ Calculated as oxypeucedanin.

**Table 6:** Antimicrobial activity of *P. trifida* essential oils and positive controls (mg/mL).

Essential Oil/Positive Control	Root		Leaf		Stem		Fruit		E211		E224		Standard Drugs ^1^	
Bacteria	MIC	MBC	MIC	MBC	MIC	MBC	MIC	MBC	MIC	MBC	MIC	MBC	MIC	MBC
*Staphylococcus aureus* (ATCC 11632)	0.20	0.39	0.78	1.56	0.20	0.39	6.25	12.5	4.00	4.00	1.00	1.00	0.1	0.2
*Bacillus cereus* (clinical isolate)	0.20	0.39	0.78	1.56	0.20	0.39	0.78	1.56	0.50	0.50	2.00	4.00	0.025	0.05
*Listeria monocytogenes* (NCTC 7973)	3.13	6.25	3.13	6.25	1.56	3.13	6.25	12.5	1.00	2.00	0.50	1.00	0.15	0.3
*Salmonella* Typhimurium (ATCC 13311)	1.56	3.13	1.56	3.13	0.78	1.56	3.13	6.25	1.00	2.00	1.00	1.00	0.1	0.2
*Escherichia coli* (ATCC 25922)	6.25	12.5	1.56	3.13	1.56	3.13	6.25	12.5	1.00	2.00	0.50	1.00	0.1	0.2
*Enterobacter cloacae* (ATCC 35030)	1.56	3.13	1.56	3.13	1.56	3.13	6.25	12.5	2.00	4.00	0.50	0.50	0.025	0.05
Fungi	MIC	MFC	MIC	MFC	MIC	MFC	MIC	MFC	MIC	MFC	MIC	MFC	MIC	MFC
*Aspergillus fumigatus* (ATCC 9197)	0.10	0.20	0.39	0.78	0.20	0.39	0.20	0.39	1.00	2.00	1.00	1.00	0.025	0.05
*Aspergillus versicolor* (ATCC 11730)	0.78	1.56	0.78	1.56	0.39	0.78	0.78	1.56	2.00	4.00	1.00	1.00	0.006	0.0125
*Aspergillus niger* (ATCC 6275)	0.20	0.39	0.39	0.78	0.20	0.39	0.39	0.78	1.00	2.00	1.00	1.00	0.10	0.20
*Penicillium funiculosum* (ATCC 36839)	0.10	0.20	0.10	0.20	0.10	0.20	0.10	0.20	1.00	2.00	0.50	0.50	0.0125	0.025
*Penicillium verrucosum* (food isolate)	0.10	0.20	0.10	0.20	0.10	0.20	0.10	0.20	2.00	4.00	1.00	1.00	0.06	0.0125
*Trichoderma harzianum* (TH-IS005-12)	0.10	0.20	0.10	0.20	0.20	0.39	0.39	0.39	1.00	2.00	0.50	0.50	0.2	0.4
*Candida albicans* (ATCC 10231)	1.56	3.13	1.56	3.13	3.13	6.25	1.56	3.13	1.00	2.00	1.00	2.00	0.0125	0.025

^1^ Standard drugs: streptomycin (antibacterial activity) and ketoconazole (antifungal activity).

**Table 7:** Antibiofilm activity of *P. trifida* essential oils (%).

Essential oils	Root			Leaf			Stem			Fruit		
Bacteria	MIC	½MIC	¼MIC	MIC	½MIC	¼MIC	MIC	½MIC	¼MIC	MIC	½MIC	¼MIC
*Staphylococcus aureus*	39.4 ± 4.6 ^c^	37.7 ± 5.3 ^c^	24.2 ± 2.7	55.4 ± 3.8 ^d^	28.2 ± 2.9 ^b^	NA^1^	22.6 ± 4.2 ^b^	35.5 ± 4.3 ^c^	NA	14.9 ± 1.1 ^a^	2.5 ± 0.6 ^a^	NA
*Listeria monocytogenes*	79.3 ± 6.6 ^b^	80.2 ± 7.2 ^c^	21 ± 2.8 ^b^	87.1 ± 4.1 ^c^	82.6 ± 6.4 ^c^	50.3 ± 1.1 ^c^	17.1 ± 1.1 ^a^	32.4 ± 2.4 ^b^	22.9 ± 1.5 ^b^	89.1 ± 1.7 ^c^	15.7 ± 4.1 ^a^	2.8 ± 0.4 ^a^
*Escherichia coli*	51.1 ± 3.3 ^c^	27.3 ± 2.7 ^b^	15.0 ± 1.0 ^a^	62.5 ± 5.4 ^d^	13.1 ± 1.5 ^a^	NA	2.3 ± 0.1 ^a^	NA	NA	45.2 ± 6.8 ^b^	46.3 ± 6.2 ^c^	27.2 ± 3.2 ^b^
Fungus	MIC	½MIC	¼MIC	MIC	½MIC	¼MIC	MIC	½MIC	¼MIC	MIC	½MIC	¼MIC
*Candida albicans*	2.5 ± 0.3 ^b^	NA	NA	1.2 ± 0.1 ^a^	NA	NA	NA	NA	NA	3.3 ± 0.1 ^c^	NA	NA

^1^ NA, no activity; different letters in each line indicate significant differences (*p* < 0.05).

**Table 8:** Antimicrobial activity of *P. trifida* MFDEs and positive controls (mg/mL).

MFDE/Positive Control	Root		Fruit		Streptomycin		Ketoconazole	
Bacteria	MIC	MBC	MIC	MBC	MIC	MBC	MIC	MBC
*Staphylococcus aureus* (ATCC 11632)	1	2	2	4	0.1	0.2	-	-
*Bacillus cereus* (clinical isolate)	0.125	0.25	0.125	0.25	0.025	0.05	-	-
*Listeria monocytogenes* (NCTC 7973)	1	2	1	2	0.15	0.3	-	-
*Salmonella* Typhimurium (ATCC 13311)	1	2	1	2	0.1	0.2	-	-
*Escherichia coli* (ATCC 25922)	4	8	4	8	0.1	0.2	-	-
*Enterobacter cloacae* (ATCC 35030)	4	8	2	4	0.025	0.05	-	-
Fungi	MIC	MFC	MIC	MFC	MIC	MFC	MIC	MFC
*Aspergillus fumigatus* (ATCC 9197)	0.25	0.5	0.25	0.5	-	-	0.025	0.05
*Aspergillus versicolor* (ATCC 11730)	0.5	1	0.5	1	-	-	0.006	0.0125
*Aspergillus niger* (ATCC 6275)	0.0625	0.125	0.0625	0.125	-	-	0.10	0.20
*Penicillium funiculosum* (ATCC 36839)	0.5	1	0.5	1	-	-	0.0125	0.025
*Penicillium verrucosum* var. *cyclopium* (food isolate)	1	2	1	2	-	-	0.06	0.0125
*Trichoderma harzianum* (TH-IS005-12)	1	2	1	2	-	-	0.2	0.4
*Candida albicans* (ATCC 10231)	1	2	1	2	-	-	0.0125	0.025

**Table 9:** Antibiofilm activity of *P. trifida* MFDEs (%).

MFDE	Root			Fruit		
Bacteria	MIC	½MIC	¼MIC	MIC	½MIC	¼MIC
*Staphylococcus aureus* (ATCC 11632)	2.7 ± 1.6	NA ^1^	NA	NA	28.3 ± 3.7	NA
*Listeria monocytogenes* (NCTC 7973)	3.2 ± 3.4	15.2 ± 4.3	27.6 ± 2.2 ^b^	NA	NA	1.3 ± 0.9 ^a^
*Escherichia coli* (ATCC 25922)	NA	NA	NA	2.3 ± 1.8	NA	NA
Fungus	MIC	½MIC	¼MIC	MIC	½MIC	¼MIC
*Candida albicans* (ATCC 10231)	53.5 ± 6.0 ^a^	32.3 ± 5.2 ^b^	26.8 ± 10.2 ^b^	48.6 ± 5.1 ^a^	23.0 ± 3.9 ^a^	14.3 ± 7.9 ^a^

^1^ NA, no activity; different letters in each line indicate significant differences (*p* < 0.05).

**Table 10:** Effects of *P. trifida* MFDEs on *C. albicans* EPS production inhibition (%).

MFDE	MIC	½MIC	¼MIC
Root	23.7 ± 8.1 ^1,a^	14.4 ± 5.4 ^a^	33.7 ± 6.1 ^b^
Fruit	31.5 ± 5.1 ^b^	21.5 ± 7.0 ^b^	13.1 ± 3.7 ^a^

^1^ Different letters in each column indicate significant differences (*p* < 0.05).

## Data Availability

Data are contained within the article and supplementary material.

## Supplementary Materials

The following supporting information can be downloaded at: https://www.mdpi.com/article/10.3390/antibiotics13010041/s1, Figure S1: *Prangos trifida* aerial parts in fruit (A and B), fruits (C-F) and dried leaf (G). Sićevo Gorge (Kusača), Serbia (2020).; Table S1: Regression equations, *r*^2^, limits of detection (LOD) and quantification (LOQ) of the compounds.

## References

[1] Stojković D., Petrović J., Carević T., Soković M., Liaras K. (2023). Synthetic and Semisynthetic Compounds as Antibacterials Targeting Virulence Traits in Resistant Strains: A Narrative Updated Review. Antibiotics.

[2] Ivanov M., Ćirić A., Stojković D. (2022). Emerging Antifungal Targets and Strategies. Int. J. Mol. Sci..

[3] Chassagne F., Samarakoon T., Porras G., Lyles J.T., Dettweiler M., Marquez L., Salam A.M., Shabih S., Farrokhi D.R., Quave C.L. (2021). A Systematic Review of Plants With Antibacterial Activities: A Taxonomic and Phylogenetic Perspective. Front. Pharmacol..

[4] Kostić M., Ivanov M., Markovic T., Sanković Babić S., Barros L., Calhelha R., Sokovic M., Ciric A. (2022). An in vitro study of the *Origanum minutiflorum* O. Schwarz & P. H. Davis and *Coriandrum sativum* L. essential oils as chronic tonsillitis therapeutics: Antibacterial, antibiofilm, antioxidant, and cytotoxic activities. J. Essent. Oil Res..

[5] Plants of the World Online. https://powo.science.kew.org.

[6] Mottaghipisheh J., Kiss T., Tóth B., Csupor D. (2020). The *Prangos* genus: A comprehensive review on traditional use, phytochemistry, and pharmacological activities. Phytochem. Rev..

[7] Bruno M., Ilardi V., Lupidi G., Quassinti L., Bramucci M., Fiorini D., Venditti A., Maggi F. (2019). The nonvolatile and volatile metabolites of *Prangos ferulacea* and their biological properties. Planta Med..

[8] Badalamenti N., Maresca V., Di Napoli M., Bruno M., Basile A., Zanfardino A. (2022). Chemical composition and biological activities of *Prangos ferulacea* essential oils. Molecules.

[9] Azarkish P., Moghaddam M., Ghasemi Pirbalouti A., Khakdan F. (2021). Variability in the essential oil of different wild populations of *Prangos platychlaena* collected from Southwestern Iran. Plant Biosyst..

[10] Zengin G., Mahomoodally M.F., Yıldıztugay E., Jugreet S., Khan S.U., Dall’Acqua S., Mollica A., Bouyahya A., Montesano D. (2022). Chemical composition, biological activities and in silico analysis of essential oils of three endemic *Prangos* species from Turkey. Molecules.

[11] Karahisar E., Köse Y.B., İşcan G., Kurkcuoglu M., Tugay O. (2022). Chemical Composition and Anticandidal Activity of Essential Oils Obtained from Different Parts of *Prangos heyniae* H. Duman & MF Watson. Rec. Nat. Prod..

[12] Sevin G., Alan E., Demir S., Albayrak G., Demiroz T., Yetik-Anacak G., Baykan S. (2022). Comparative evaluation of relaxant effects of three *Prangos* species on mouse corpus cavernosum: Chemical characterization and the relaxant mechanisms of action of *P. pabularia* and (+)-oxypeucedanin. J. Ethnopharmacol..

[13] Tan N., Yazıcı-Tütüniş S., Bilgin M., Tan E., Miski M. (2017). Antibacterial Activities of Pyrenylated Coumarins from the Roots of *Prangos hulusii*. Molecules.

[14] Zengin G., Sinan K.I., Ak G., Mahomoodally M.F., Paksoy M.Y., Picot-Allain C., Glamočlija J., Soković M., Jekő J., Cziáky Z. (2020). Chemical profile, antioxidant, antimicrobial, enzyme inhibitory, and cytotoxicity of seven Apiaceae species from Turkey: A comparative study. Ind. Crops Prod..

[15] Dall’Acqua S., Sut S., Zengin G., Peron G., Elbasan F., Yildiztugay E., Bibi Sadeer N., Mahomoodally M.F. (2022). Phytochemical screening, antioxidant, and enzyme inhibitory properties of three *Prangos* species (*P. heyniae*, *P. meliocarpoides* var. *meliocarpoides*, and *P. uechtritzii*) depicted by comprehensive LC-MS and multivariate data analysis. Antioxidants.

[16] Nosrati M., Ranjbar R. (2022). Investigation of the antibacterial and biofilm inhibitory activities of *Prangos acaulis* (DC.) Bornm in nanoparticulated formulation. Nanotechnology.

[17] Sarghaleh S.J., Behbahani B.A., Hojjati M., Vasiee A., Noshad M. (2023). Evaluation of the constituent compounds, antioxidant, anticancer, and antimicrobial potential of *Prangos ferulacea* plant extract and its effect on *Listeria monocytogenes* virulence gene expression. Front. Microbiol..

[18] Herrnstadt I., Heyn C.C. (1977). A monographic study of the genus *Prangos* (Umbelliferae). Boissiera.

[19] Niketić M., Stevanović V. (1999). *Cachrys alpina* L.. The Red Data Book of Flora of Serbia. Extinct and Critically Endangered Taxa.

[20] Hand R. (2011). Apiaceae. Euro+Med Plantbase—The Information Resource for Euro-Mediterranean Plant Diversity.

[21] Lyskov D.F. (2015). Systematics of the Genus *Prangos* (Umbelliferae, Apioideae) and Related Taxa: Comparison of Morphological-Anatomic and Molecular Data. Ph.D. Thesis.

[22] Lyskov D., Samigullin T., Oskolski A., Nuraliev M., Tilney P. (2017). European *Prangos* species complexes: When classic morphological features are not enough to distinguish similar species. Abstract Book, Proceedings of the IX Apiales Symposium, The Gold Coast Marina Club, Guangzhou, China, 31 July–2 August 2017.

[23] Baser K.H.C., Demirci B., Akalin E., Özhatay N. (2004). Composition of the Essential Oil of *Cachrys alpine* Bieb. J. Essent. Oil Res..

[24] Palá-Paül J., Velasco-Negueruela A., Pérez-Alonso J., Maqueda J., Sanz J. (2004). Volatile oil Constituents from Different Parts of *Cachrys trifida* L.. J. Essent. Oil Res..

[25] Korotkov O.I., Shevchuk O.M., Shatko V.G., Timashova L.A., Feskov S.A. (2018). Some biochemical characteristics of *Prangos trifida* (Mill.) Herrnst. & Heyn. Bull. State Nikit. Botan. Gard..

[26] Di Napoli M., Castagliuolo G., Badalamenti N., Vaglica A., Ilardi V., Varcamonti M., Bruno M., Zanfardino A. (2022). Chemical composition, antimicrobial and antioxidant activities of the essential oil of Italian *Prangos trifida* (Mill.) Herrnst. & Heyn. Nat. Prod. Res..

[27] Maresca V., Badalamenti N., Ilardi V., Bruno M., Basile A. (2023). The Antioxidant Properties and Protective Capacity of *Prangos trifida* and *Cachrys cristata* Essential Oils against Cd Stress in *Lunularia cruciata* and *Brassica napus*. Antioxidants.

[28] Abad M.J., De Las Heras B., Silván A.M., Pascual R., Bermejo P., Rodriguez B., Villar A.M. (2001). Effects of furocoumarins from *Cachrys trifida* on some macrophage functions. J. Pharm. Pharmacol..

[29] Adams R.P. (2017). Identification of Essential Oil Components by Gas Chromatography/Mass Spectrometry.

[30] Khalighi-Sigaroodi F., Hadjiakhoondi A., Shafiee A., Mozaffarian V.A., Shahverdi A.R., Alavi S.H. (2006). Phytochemical analysis of *Ferulogo bernardii* Tomk & M. Pimen. DARU J. Pharm. Sci..

[31] Wei Y., Ito Y. (2006). Preparative isolation of imperatorin, oxypeucedanin and isoimperatorin from traditional Chinese herb “bai zhi” *Angelica dahurica* (Fisch. ex Hoffm) Benth. et Hook using multidimensional high-speed counter-current chromatography. J. Chromatogr. A.

[32] Sajjadi S.E., Jamali M., Shokoohinia Y., Abdi G., Shahbazi B., Fattahi A. (2015). Antiproliferative evaluation of terpenoids and terpenoid coumarins from *Ferulago macrocarpa* (Fenzl) Boiss. fruits. Pharmacogn. Res..

[33] Ušjak L.J., Drobac M.M., Niketić M.S., Petrović S.D. (2018). Chemosystematic Significance of Essential Oil Constituents and Furanocoumarins of Underground Parts and Fruits of Nine *Heracleum* L. Taxa from Southeastern Europe. Chem. Biodivers..

[34] Kiyonga A.N., Hong G., Kim H.S., Suh Y.G., Jung K. (2021). Facile and Rapid Isolation of Oxypeucedanin Hydrate and Byakangelicin from *Angelica dahurica* by Using [Bmim] Tf2N Ionic Liquid. Molecules.

[35] Frérot E., Decorzant E. (2004). Quantification of Total Furocoumarins in Citrus Oils by HPLC Coupled with UV, Fluorescence, and Mass Detection. J. Agric. Food Chem..

[36] Dewick P.M. (2009). Medicinal Natural Products: A Biosynthetic Approach.

[37] Kusano M., Kobayashi M., Iizuka Y., Fukushima A., Saito K. (2016). Unbiased profiling of volatile organic compounds in the headspace of *Allium* plants using an in-tube extraction device. BMC Res. Notes.

[38] Skalicka-Woźniak K., Głowniak K. (2012). Pressurized liquid extraction of coumarins from fruits of *Heracleum leskowii* with application of solvents with different polarity under increasing temperature. Molecules.

[39] Bagci E. (2007). Fatty acids and tocochromanol patterns of some Turkish Apiaceae (Umbelliferae) plants; a chemotaxonomic approach. Acta Bot. Gallica.

[40] Küçükboyacι N., Ayaz F., Adιgüzel N., Bani B., Gören A.C. (2016). Fatty Acid Methyl Ester Composition of Some Turkish Apiaceae Seed Oils: New Sources for Petroselinic Acid. Nat. Prod. Commun..

[41] Saini R.K., Song M.H., Yu J.W., Shang X., Keum Y.S. (2021). Phytosterol Profiling of Apiaceae Family Seeds Spices Using GC-MS. Foods.

[42] Kuznetsova G.A., Belenovskaya L.M. (1966). Prantschimgin—A new coumarin from the roots of *Prangos tschimganica*. Chem. Nat. Compd..

[43] Basilico M.Z., Basilico J.C. (1999). Inhibitory effects of some spice essential oils on *Aspergillus ochraceus* NRRL 3174 growth and ochratoxin A production. Lett. Appl. Microbiol..

[44] Bayman P., Baker J.L., Doster M.A., Michailides T.J., Mahoney N.E. (2002). Ochratoxin production by the *Aspergillus ochraceus* group and *Aspergillus alliaceus*. Appl. Environ. Microbiol..

[45] Mazaheri T., Cervantes-Huamán B.R., Bermúdez-Capdevila M., Ripolles-Avila C., Rodríguez-Jerez J.J. (2021). *Listeria monocytogenes* biofilms in the food industry: Is the current hygiene program sufficient to combat the persistence of the pathogen?. Microorganisms.

[46] Zhao L., Poh C.N., Wu J., Zhao X., He Y., Yang H. (2022). Effects of electrolysed water combined with ultrasound on inactivation kinetics and metabolite profiles of *Escherichia coli* biofilms on food contact surface. Innov. Food Sci. Emerg. Technol..

[47] Idrees M., Sawant S., Karodia N., Rahman A. (2021). *Staphylococcus aureus* biofilm: Morphology, genetics, pathogenesis and treatment strategies. Int. J. Environ. Res. Public Health.

[48] Villa K., Sopha H., Zelenka J., Motola M., Dekanovsky L., Beketova D.C., Macak J.M., Ruml T., Pumera M. (2022). Enzyme-photocatalyst tandem microrobot powered by urea for *Escherichia coli* biofilm eradication. Small.

[49] Rios J.L., Recio M.C. (2005). Medicinal plants and antimicrobial activity. J. Ethnopharmacol..

[50] Miladi H., Zmantar T., Kouidhi B., Al Qurashi Y.M., Bakhrouf A., Chaabouni Y., Mahdouani K., Chaieb K. (2017). Synergistic effect of eugenol, carvacrol, thymol, *p*-cymene and *γ*-terpinene on inhibition of drug resistance and biofilm formation of oral bacteria. Microb. Pathog..

[51] Reichling J. (2020). Anti-biofilm and Virulence Factor-Reducing Activities of Essential Oils and Oil Components as a Possible Option for Bacterial Infection Control. Planta Med..

[52] Purkait S., Bhattacharya A., Bag A., Chattopadhyay R.R. (2020). Evaluation of antibiofilm efficacy of essential oil components β-caryophyllene, cinnamaldehyde and eugenol alone and in combination against biofilm formation and preformed biofilms of *Listeria monocytogenes* and *Salmonella typhimurium*. Lett. Appl. Microbiol..

[53] Kerekes E.B., Deák É., Takó M., Tserennadmid R., Petkovits T., Vágvölgyi C., Krisch J. (2013). Anti-biofilm forming and anti-quorum sensing activity of selected essential oils and their main components on food-related micro-organisms. J. Appl. Microbiol..

[54] Khan F., Tabassum N., Jeong G.J., Jung W.K., Kim Y.M. (2023). Inhibition of Mixed Biofilms of *Candida albicans* and *Staphylococcus aureus* by β-Caryophyllene-Gold Nanoparticles. Antibiotics.

[55] Khoury M., El Beyrouthy M., Eparvier V., Ouaini N., Stien D. (2018). Chemical diversity and antimicrobial activity of the essential oils of four Apiaceae species growing wild in Lebanon. J. Essent. Oil Res..

[56] Yousefi K., Hamedeyazdan S., Hodaei D., Lotfipour F., Baradaran B., Orangi M., Fathiazad F. (2017). An in vitro ethnopharmacological study on *Prangos ferulacea*: A wound healing agent. Bioimpacts.

[57] Özek G., Özek T., İşcan G., Başer K.H.C., Hamzaoglu E., Duran A. (2007). Comparison of hydrodistillation and microdistillation methods for the analysis of fruit volatiles of *Prangos pabularia* Lindl., and evaluation of its antimicrobial activity. S. Afr. J. Bot..

[58] Brusotti G., Ibrahim M.F., Dentamaro A., Gilardoni G., Tosi S., Grisoli P., Dacarro C., Guglielminetti M.L., Hussain F.H.S., Caccialanza G. (2013). Chemical composition and antimicrobial activity of the volatile fractions from leaves and flowers of the wild Iraqi Kurdish plant *Prangos peucedanifolia* Fenzl. Chem. Biodivers..

[59] Uzel A., Dirmenci T., Çelik A., Arabaci T. (2006). Composition and antimicrobial activity of *Prangos platychlaena* and *P. uechtritzii*. Chem. Nat. Compd..

[60] Latgé J.P., Chamilos G. (2019). *Aspergillus fumigatus* and Aspergillosis in 2019. Clin. Microbiol. Rev..

[61] Karakaya S., Şimşek D., Özbek H., Güvenalp Z., Altanlar N., Kazaz C., Kiliç C.S. (2019). Antimicrobial Activities of Extracts and Isolated Coumarins from the Roots of Four *Ferulago* Species Growing in Turkey. Iran. J. Pharm. Res..

[62] Ulubelen A., Topcu G., Tan N., Ölçal S., Johansson C., Üçer M., Birman H., Tamer Ş. (1995). Biological activities of a Turkish medicinal plant, *Prangos platychlaena*. J. Ethnopharmacol..

[63] Mileski K.S., Trifunović S.S., Ćirić A.D., Šakić Z.M., Ristić M.S., Todorović N.M., Matevski V.S., Marin P.D., Tešević V.V., Džamić A.M. (2017). Research on Chemical Composition and Biological Properties Including Antiquorum Sensing Activity of *Angelica pancicii* Vandas Aerial Parts and Roots. J. Agric. Food Chem..

[64] Leite M.C.A., De Brito Bezerra A.P., De Sousa J.P., De Oliveira Lima E. (2015). Investigating the antifungal activity and mechanism(s) of geraniol against *Candida albicans* strains. Med. Mycol..

[65] Ali S., Sudha K.G., Thirumalaivasan N., Ahamed M., Pandiaraj S., Rajeswari V.D., Vinayagam Y., Thiruvengadam M., Govindasamy R. (2023). Green Synthesis of Magnesium Oxide Nanoparticles by Using *Abrus precatorius* Bark Extract and Their Photocatalytic, Antioxidant, Antibacterial, and Cytotoxicity Activities. Bioengineering.

[66] Gayathiri E., Prakash P., Kumaravel P., Jayaprakash J., Ragunathan M.G., Sankar S., Pandiaraj S., Thirumalaivasan N., Thiruvengadam M., Govindasamy R. (2023). Computational approaches for modeling and structural design of biological systems: A comprehensive review. Prog. Biophys. Mol. Biol..

[67] Pemán J., Cantón E., Espinel-Ingroff A. (2009). Antifungal drug resistance mechanisms. Expert Rev. Anti-Infect. Ther..

[68] EDQM (2023). European Pharmacopoeia 11.0.

[69] Arsenijević J., Marković J., Šoštarić I., Ražić S. (2013). A chemometrics as a powerful tool in the elucidation of the role of metals in the biosynthesis of volatile organic compounds in Hungarian thyme samples. Plant Physiol. Biochem..

[70] Ušjak L., Sofrenić I., Tešević V., Drobac M., Niketić M., Petrović S. (2019). Fatty acids, sterols, and triterpenes of the fruits of 8 *Heracleum* taxa. Nat. Prod. Commun..

[71] Tang J.J., Zhao N., Gao Y.Q., Han R., Wang X.Y., Tian J.M., Gao J.M. (2021). Phytosterol profiles and iridoids of the edible *Eucommia ulmoides* Oliver seeds and their anti-inflammatory potential. Food Biosci..

[72] Kostić M., Smiljković M., Petrović J., Glamočlija J., Barros L., Ferreira I.C.F.R., Ćirić A., Soković M. (2017). Chemical, nutritive composition and a wide range of bioactive properties of honey mushroom: *Armillaria mellea* (Vahl: Fr.) Kummer. Food Funct..

[73] Ivanov M., Kostić M., Stojković D., Soković M. (2022). Rosmarinic acid–Modes of antimicrobial and antibiofilm activities of common plant polyphenol. S. Afr. J. Bot..

[74] Ivanov M., Gašić U., Stojković D., Kostić M., Mišić D., Soković M. (2021). New Evidence for *Artemisia absinthium* L. Application in Gastrointestinal Ailments: Ethnopharmacology, Antimicrobial Capacity, Cytotoxicity, and Phenolic Profile. Evid. Based Complement. Alternat. Med..

