# Supplementary material for: Chemical Analysis and Investigation of Antimicrobial and Antibiofilm Activities of Prangos trifida (Apiaceae)
Source: Antibiotics (Basel). 2024 Jan 1;13(1):41. doi: 10.3390/antibiotics13010041 (PMC10812483; doi:10.3390/antibiotics13010041)
Supplement: Supplementary file 1 [file antibiotics-13-00041-s001.zip › Figure S1.pdf]

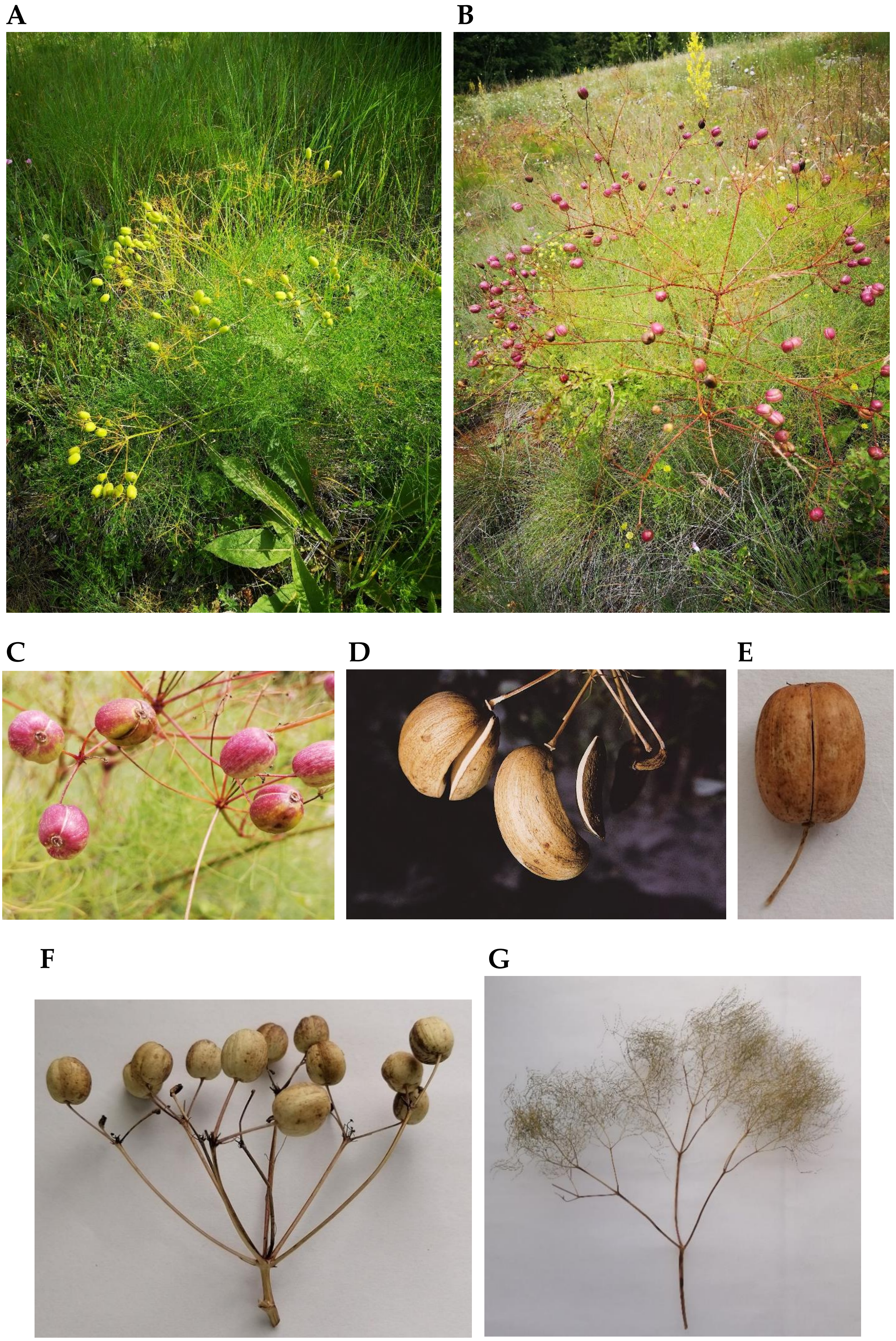

Figure S1. *Prangos trifida* aerial parts in fruit (A and B), fruits (C-F) and dried leaf (G). Sićevo Gorge (Kusača), Serbia (2020).
