# Supplementary material for: Chemical Analysis and Investigation of Antimicrobial and Antibiofilm Activities of Prangos trifida (Apiaceae)
Source: Antibiotics (Basel). 2024 Jan 1;13(1):41. doi: 10.3390/antibiotics13010041 (PMC10812483; doi:10.3390/antibiotics13010041)
Supplement: Supplementary file 1 [file antibiotics-13-00041-s001.zip › Table S1.pdf]

Table S1. Regression equations, r^2, limits of detection (LOD) and quantification (LOQ) of the compounds.

Compound	Regression equation	r^2	LOD, µg	LOQ, µg
Imperatorin (**7**) [1]	y=14013.0048x+39.9148	0.9998	0.0281	0.0853
Imperatorin (**7**) [2]	y=4E+08x+4E+06	0.9938	0.1101	0.3337
Oxypeucedanin (**6**) [1]	y=3354.0961x+2.3841	0.9996	0.0833	0.2525
Prantschimgin (**10**) [3]	y=4271.7028x+269.9534	0.9987	0.1646	0.4988

[1] Obtained using peak areas recorded on DAD at 250 nm. [2] Obtained using peak areas recorded on MSD in Single Ion Monitoring (SIM) of *m/z* 271.1 mode. [3] Obtained using peak areas recorded on DAD at 350 nm.
